# Ecophenotypic Variation and Developmental Instability in the Late Cretaceous Echinoid *Micraster brevis* (Irregularia; Spatangoida)

**DOI:** 10.1371/journal.pone.0148341

**Published:** 2016-02-05

**Authors:** Nils Schlüter

**Affiliations:** Georg-August University of Göttingen, Geoscience Centre, Department of Geobiology, Goldschmidtstr. 3, 37077, Göttingen, Germany; Naturhistoriska riksmuseet, SWEDEN

## Abstract

The Late Cretaceous echinoid genus *Micraster* (irregular echinoids, Spatangoida) is one of the most famous examples of a continuous evolutionary lineage in invertebrate palaeontology. The influence of the environment on the phenotype, however, was not tested so far. This study analyses differences in phenotypical variations within three populations of *Micraster* (*Gibbaster*) *brevis* from the early Coniacian, two from the Münsterland Cretaceous Basin (Germany) and one from the North Cantabrian Basin (Spain). The environments of the Spanish and the German sites differed by their sedimentary characteristics, which are generally a crucial factor for morphological adaptations in echinoids. Most of the major phenotypical variations (position of the ambitus, periproct and development of the subanal fasciole) among the populations can be linked to differences in their host sediments. These phenotypic variations are presumed to be an expression of phenotpic plasticiy, which has not been considered in *Micraster* in previous studies. Two populations (Erwitte area, Germany; Liencres area, Spain) were tested for stochastic variation (fluctuating asymmetry) due to developmental instability, which was present in all studied traits. However, differences in the amount of fluctuating asymmetry between both populations were recognised only in one trait (amount of pore pairs in the anterior paired petals). The results strengthen previous assumptions on ecophenotypic variations in *Micraster*.

## Introduction

The environment plays a principal role in adaptive evolution by shaping through natural selection the means of population phenotypes and by evoking phenotypic variation without altering the genetic background, e.g. through uncovering cryptic genetic variation (decanalization), or phenotypic plasticity [1–4]. Phenotypic plasticity, however, is a property of a genotype and, thus, its norm of reaction is influenced by genetic variation as well [5]. The developmental origins of observed phenotypic variation are often open to debate in palaeontological studies, reasoned in the complex interplay of genes and environment. Typically, the fossil record provides, to some extent, only the factor environment in this equation. However, to decipher patterns in development, evolution and speciation in deep time, it is meaningful to address these mechanisms in studies for fossil taxa as well [6]. However, another source of phenotypic variation—developmental instability—is easy to explore but received limited attention by palaeontologists. As a consequence of developmental instability, environmental or genetic stressors perturb developmental pathways (within populations of similar genotypes and stable environments) and lead to an increase in phenotypic variation [7, 8]. Developmental instability can be measured by fluctuating asymmetry, random (subtle) deviations from perfect symmetry, as the genes on both sides of a symmetric organism are identical, barring somatic mutations [9]. Developmental stability, on the other hand, is the ability to buffer the development against those perturbations. Developmental instability has ample origins and the processes leading to it are still not well understood (reviewed in [10, 11]). Stochastic gene expression contributes to developmental noise [12, 13], caused, for example, by environmental stressors, such as pollution, or mutations that reduce the activity of a gene.

**Fig 1:**
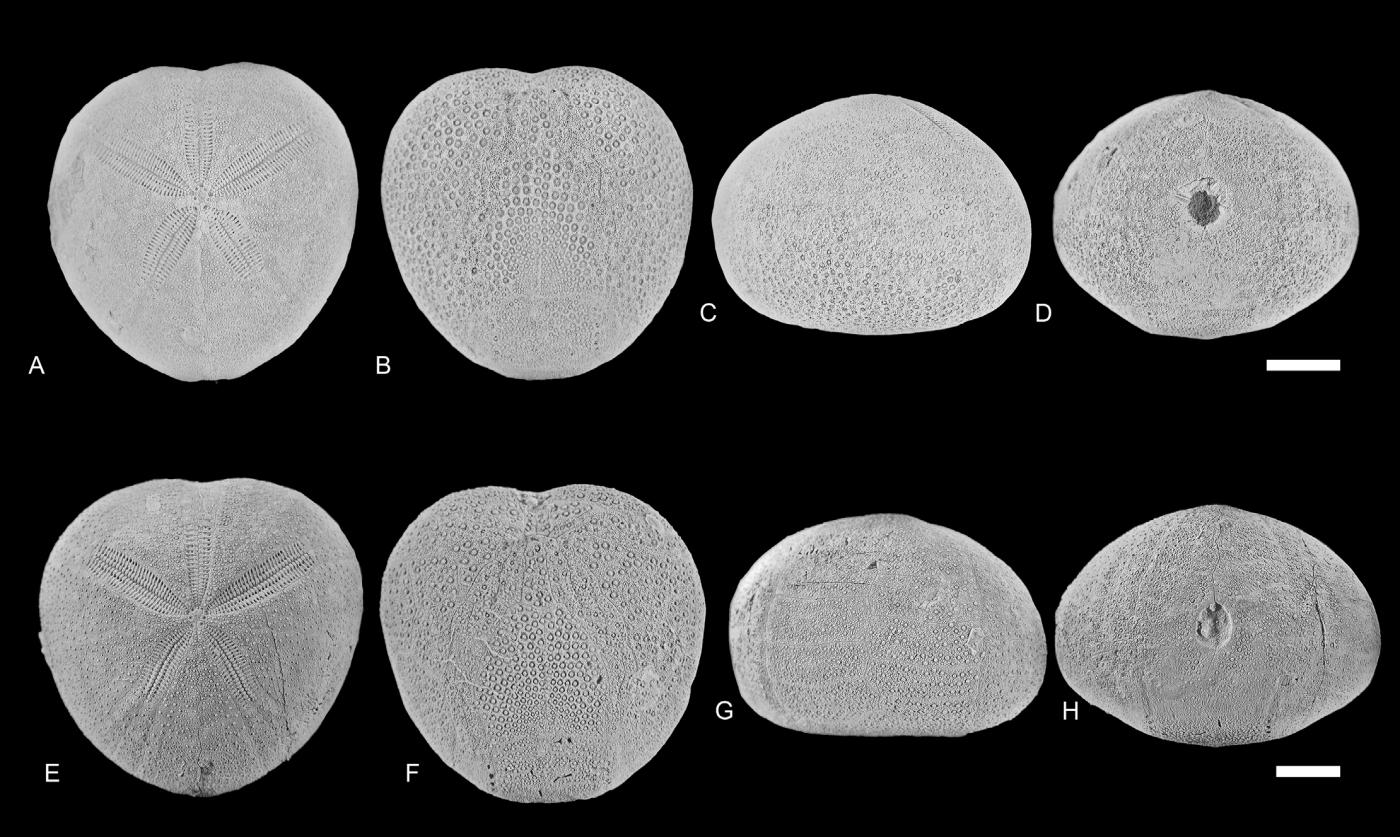
*Micraster brevis*. (A-D) Specimen from the Liencres area, Spain (MB.E.8251) in apical, oral, lateral and posterior views. (E-H) Specimen from the Erwitte area, Germany (GSUB E3867) in apical, oral, lateral and posterior views. Scale bar equals 1 cm.

**Fig 2:**
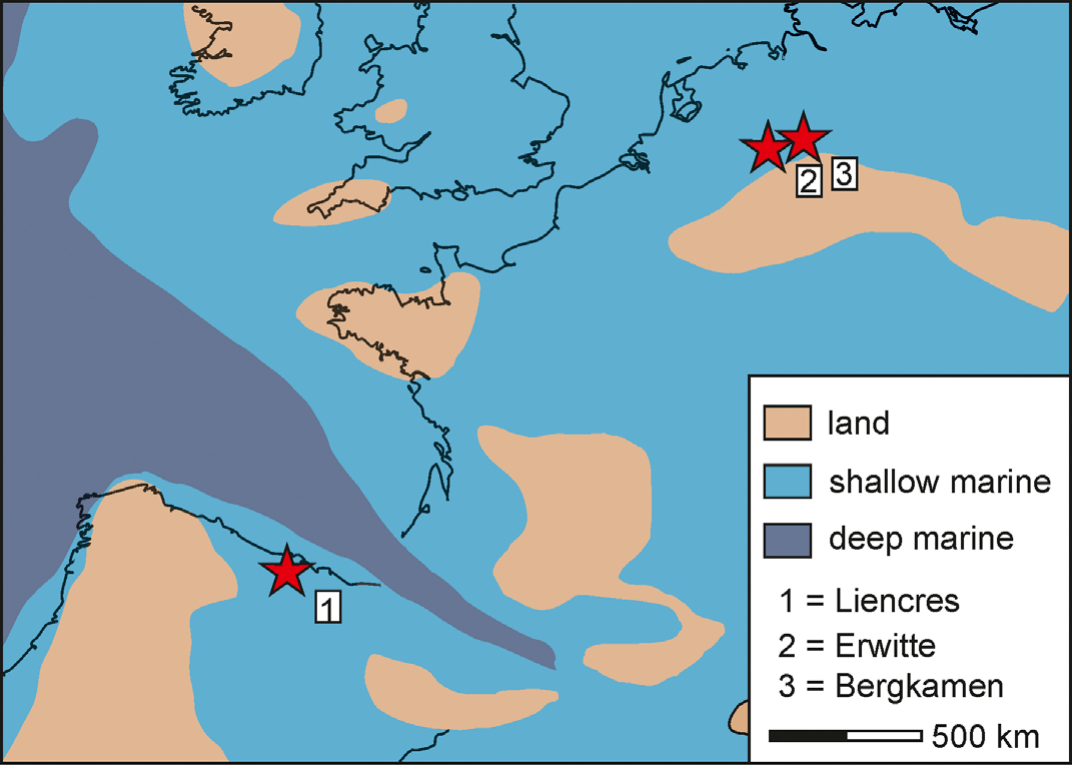
Palaeogeographic map. Simplified palaeogeographic map of western Europe during the Late Cretaceous (modified after [30]).

Phenotypic variations also cause confusion, especially for the species concept in palaeontology, exemplified by the Late Cretaceous fossil echinoid *Micraster*. According to their high variability, extensive taxonomic works resulted in vast names of species (compare [14]). *Micraster* was geographically widespread (western Asia, Europe, northern Africa [15]) and inhabited a wide spectrum of environments. Moreover, *Micraster* provides some of the most studied fossil examples in speciation (e.g. [16–20]), due to their well-traceable shifts of phenotypic variations in time. The influence of the environment on the phenotypic variation in *Micraster*, however, was only vaguely assumed [17, 18, 21], and Ernst and Seibertz [22] suggested that species of *Micraster* could have developed local ecophenotypism. However, it was assumed that the evolution in *Micraster* would reflect a step-wise increase of adaptation towards a stable environment [23]. These ideas, however, have never been tested. To analyse and discuss the influence of the environment on the phenotype of *Micraster*, three populations of *Micraster* (*Gibbaster*) *brevis* (Fig 1, following: *M*. *brevis*) were compared, which was widespread during the early Coniacian in Europe (compare [24]). Villier and colleagues [25] demonstrated that well-elongated pores in the frontal ambulacrum (associated to tube feet for gaseous exchange) in the Lower Cretaceous spatangoid *Heteraster* indicate an adaptation to warm shallow waters, which was also likely the case for *M*. *brevis*. Three populations were compared. Two samples come from the Münsterland Cretaceous Basin (MCB: Grimberg (IV) mine shaft, Erwitte area, Germany), and one comes from the North Cantabrian Basin (NCB: Liencres area, northern Spain) (Fig 2).

Palaeoenvironments can be deduced from the composition of the rocks. While the MCB is characterised by wackestones with fine siliciclastic and calcareous ooze and a low content of dispersed bioclasts (calcispheres and foraminifera) (S1A–S1C Fig), the facies of the NCB contains silty packstones with abundant and coarser-grained, reworked silici- and bioclastics (e.g. bivalves, foraminifera, hexactinellid sponges, echinoderms) (S1D and S1E Fig). More details on the lithology can be found in [26] for the NCB and [27, 28] for the MCB. Additionally, stronger sea currents and a shallower sea level can be inferred by the sediment nature (coarse grains, angular silt) for Liencres [26] in comparison to the Grimberg (IV) mine shaft and the Erwitte area (fine-grained matrix). Accordingly, a somewhat more proximal position is inferred for Liencres than for the Erwitte area, which had a more basinal position in the inner shelf basin (Münsterland Cretaceous Basin) [27, 28]. Furthermore, a somewhat higher palaeotemperature for the Liencres area can be assumed, as a consequence of its lower palaeolatitude (compare [29] for the Cenomanian age).

In order to assess the impact of the environment on *M*. *brevis*, shape variations between the populations were analysed by a geometric morphometric approach, and additional comparative semi-quantitative analyses of four further traits were conducted (ornamentation of the periplastronal ambulacrals and the interradial area of the paired petals, projection of the labrum, development of the subanal fasciole) (see Table 1). Characters which had been previously assumed to be influenced by the sedimentary environment in spatangoid taxa were included in the analyses (e.g. position of the periproct, development of the subanal fasciole, shape of the plastron, inflation of the test) [31–35], as well as traits in which modifications are traditionally regarded as being the result of a continuous process in the evolution of *Micraster* (e.g. ornamentation of the periplastronal ambulacrals and the interradial area of the paired petals, projection of the labrum, shape of the test) [16–18, 36, 37] (Table 1). In a second part, the populations were tested for the presence and differences in the level of fluctuating asymmetry (FA) to assess if both populations deviated in their degree of developmental stability. For FA analyses, morphometric (periplastronal ambulacrals, paired petals) and meristic traits (the amount of pore pairs in the anterior and posterior paired petals) were considered.

**Table 1:** Investigated traits, their function, and evolutionary significance.

**morphometric traits**	**landmarks**	**morphological function**	**evolutionary significance**
position of the apical shield	1	growth dependent in several taxa, possible related to burrowing strategy [23, 36]	shift in position from anterior to posterior [15–20, 37]
shape of the paired petals	2–9	associated pore pairs are related to gaseous exchange [38]	increase of length [15, 16, 20]
shape of the plastron (sternal plates)	10, 12, 14–19, 21–22	related to locomotion behaviour, nature of the substrate respectively [31, 35]	not considered so far
position of the periplastronal ambulcral plates	11–15, 19–23	not known	possible shift into the anterior direction, and elongation of the perilabral plate, compare [20]
depth of the anterior notch	24–26	related to feeding strategies [36]	a trend towards a deepening of the notch [36]
position of the ambitus	24–28	related to burrowing depth [32]	lowered in the *Gibbaster* branch [17, 18]
position of the widest point of the test	27–28	not known, possible related to burrowing strategy [23]	shift towards the posterior direction [15–20, 37]
position of the periproct	30	related to burrowing depth [33]	lowered in the *Gibbaster* branch [17, 18]
**non-morphometric traits**		**morphological function**	**evolutionary significance**
pore numbers in the paired petals		associated tube feet are related to gaseous exchange [38]	increase in numbers [15, 20]
structure in the interradial area in the paired petals		not known	accentuation of the interradial structure and ornamentation [15–20, 36]
structure in the periplastronal ambulcral plates		not known	accentuation of the granulation [15–20, 37]
projection of the labrum		not known, assumed to be related to a change in feeding strategy	increase of the projection of the labrum, accordingly the peristomal opening is completely covered [15–20, 37]

## Material and Methods

### Material

In total, 126 specimens were studied (see S1 File for individual collection numbers), which originate from the coastline of Liencres (northern Cantabria, Spain, 49 specimens), from the abandoned Grimberg (IV) mine shaft (27 specimens) close to Bergkamen (Westfalia, Germany), and from the vicinity of Erwitte (Westfalia, Germany, 50 specimens). The material from the Erwitte area was collected by Ekbert Seibertz (Wolfsburg, Germany) during the construction of the highway A44 in the 1970s. The specimens from the Liencres and the Erwitte areas originate from a quasi-isochronous short-term interval of 2 m thickness (equivalent to approximately 30,000 years) from the basal *Cremnoceramus crassus crassus/Cremnoceramus deformis deformis* Zone, early Coniacian (compare [26, 39]). The Grimberg material is from the lower *Cr*. *crassus crassus/Cr*. *deformis deformis* Zone, collected between sinking depths of 286 and 282 m [40], corresponding to an interval of approximately 60,000 years. Although *M*. *brevis* is very abundant in each of the studied localities, specimens are often distorted or incomplete, which limited the sample size in this study.

For the comparison of shape variations, 86 specimens in total were analysed: 14 from the Grimberg VI mine shaft, 37 from the surroundings of Erwitte, and 35 from the Liencres area. To explore the shape variability of all populations, a Principal Component Analysis (PCA) was conducted. For FA analysis, specimens that were not adequately preserved (slight deformations) were excluded, resulting in 33 specimens from Erwitte and 35 from the Liencres area. The material from the Grimberg mine shaft was insufficient in numbers to give reliable results, and omitted for this study. Semiquantitative analyses were conducted by inclusion of 121 specimens in total (46 Liencres area, 49 Erwitte area, 26 Grimberg area) to examine the development of the subanal fasciole, the variation of the interradial structure of the paired petals, and the granulation of the periplastronal area.

For analysis of the projection of the labrum, data from 96 specimens were obtained (33 Liencres area, 46 Erwitte area, 17 Grimberg area). Due to the fragile nature of the labrum tip, the extension of the labrum was not considered in the morphometric analyses, as it would have reduced the available material.

To investigate for differences in variation of the average count and variation in FA of pore pair numbers, 35 specimens were included for the Erwitte population and 36 specimens for the Liencres population. The same material was used to test for FA in pore pair numbers in the anterior and the posterior paired petals. No permits were required for the described study, which complied with all relevant regulations.

#### Institutional abbreviations

To denote the repositories of specimens illustrated and/or referred to in the text, the following abbreviations are used: (MB.E.) Museum für Naturkunde, Leibniz-Institut für Evolutions- und Biodiversitätsforschung an der Humboldt-Universität zu Berlin, Berlin, Germany; (BGR) Bundesanstalt für Geowissenschaften und Rohstoffe Berlin, Berlin, Germany; (GSUB) Geowissenschaftliche Sammlung der Universität Bremen, Bremen, Germany.

### Methods–geometric morphometrically based analyses

In order to realise 3D models of the material, specimens were mounted on a stick and positioned on a turntable, and photos were obtained from all available perspectives of the echinoids by rotating the turntable across small angles. Overview photographs were supplemented by close-ups for capturing highly detailed 3D models. For photogrammetric reconstructions resulting in digital three-dimensional models, the images were elaborated with Autodesk^®^ 123D^®^ Catch. Each 3D model (see S1 Multimedia) was reconstructed by implementing and aligning 70 2D images. This process results in a mesh, which can be exported as a textured object file that features the photographic detailed surfaces of the specimen. These files were applied for further data collection for geometric morphometric purposes. For a more detailed and practical guide for photogrammetry, it is referred to the works of Falkingham [41] and Malison and Wings [42].

**Fig 3:**
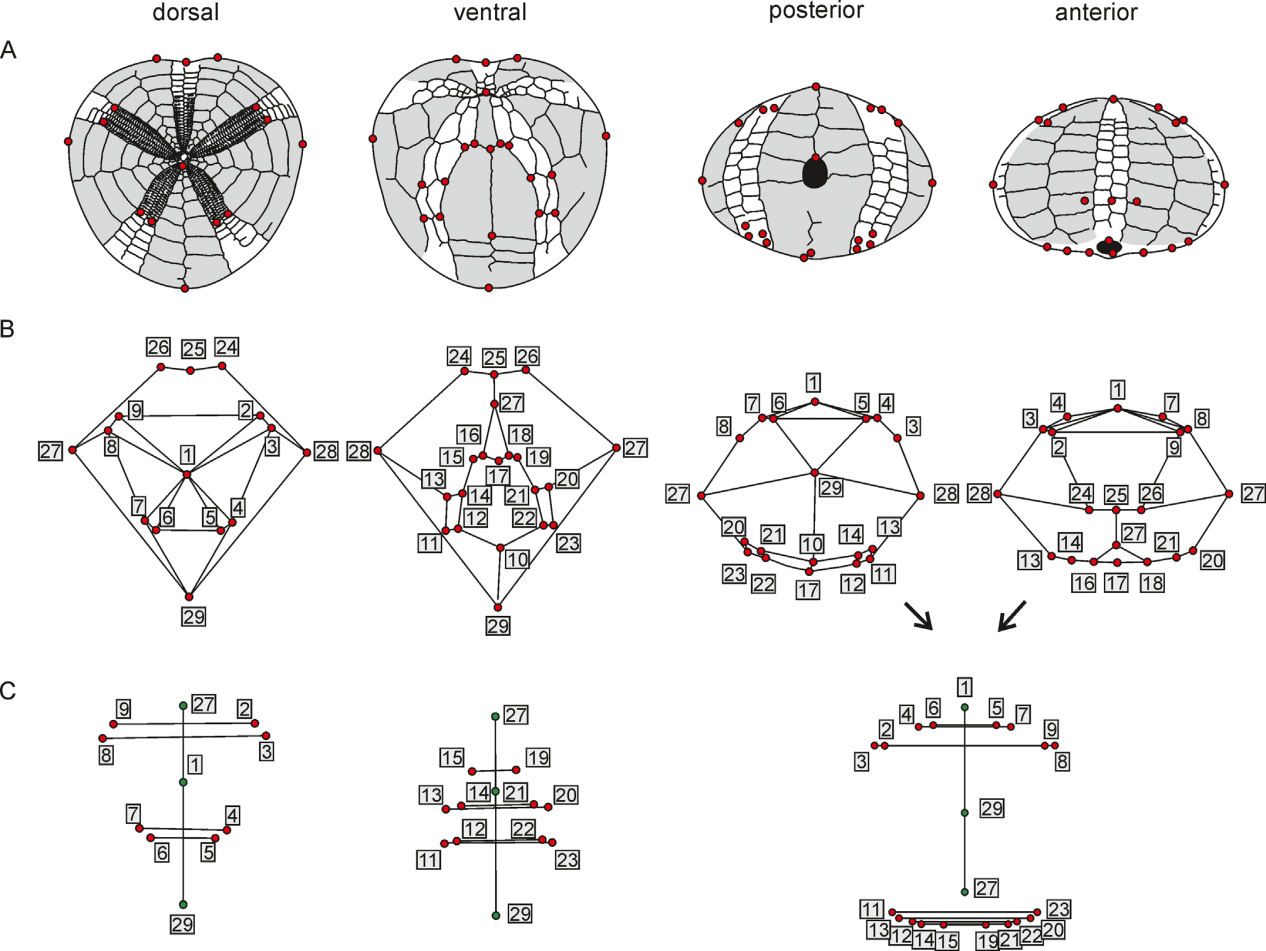
Landmark configurations. (A) Plating drawings (after specimen GSUB E3867) depicting the landmark configurations for the specific analyses. (B) Global shape variation, shown as wireframe graph. (C) Fluctuating asymmetry analysis. For FA analysis: red circles represent paired landmarks, green circles represent median landmarks.

Landmarks were digitised in the freeware MeshLab (Visual Computing Lab—ISTI—CNR; http://meshlab.sourceforge.net/) from the textured 3D models. The Cartesian coordinates were analysed with the morphometric software package MorphoJ [43]. Prior to each analysis a Procrustes fit was performed, in which the shape of each specimen was rescaled to unit centroid size, which removes information on size. Centroid size is the square root of the sum of squared distances from a configuration of landmark to the centre of the shape configuration (centroid) [44]. The rescaled shapes are then translated to the same position, and rotated to the best fitting orientation of the landmark configurations. Shape variations within the whole sample were investigated through a PCA by assessing a set of 30 landmarks (Fig 3 and S1 Multimedia).

A Procrustes ANOVA was performed to assess for the possible presence of FA and to quantify the relative amounts of shape variation in asymmetry and measurement error [45]. Because measurement error can inflate FA, it is preferred to test for its significance. The Procrustes ANOVA contains the factor sides (fixed) and individuals (random). Directional asymmetry (DA) is tested by the side factor, while FA is estimated by the individual-by-side interaction term. Occurrence of antisymmetry was estimated by examining the scatter plots of shape asymmetry for bimodality. Other asymmetric variations, e.g. DA, and antisymmetry are not suitable for measuring developmental instability as they have a genetic component and should be therefore avoided in such studies [7]. As the studied traits here have object symmetry (objects which are symmetric by themselves), tests for size dependency of FA are precluded [46]. Accordingly, only shape FA can be considered here.

A set of replicated models of each specimen was used to quantify the measurement error. For the analysis of FA, 21 landmarks, 18 paired and 3 median landmarks (Fig 3), were digitised. Only landmarks of type 1 [44] were chosen, which were precisely defined by intersections with other plate sutures, or very well locatable. The landmark configuration included the shape of the periplastronal ambulacrals and paired petals.

The Procrustes ANOVA was independently applied for the Erwitte and the Liencres populations. In both samples a Procrustes ANOVA was computed for the global shape and for each trait separately (periplastronal ambulacral plates, paired petals). Levene's test was performed to test for differences in FA among populations, which is more robust to departures from normality [7]. To visualise and investigate shape variations, a PCA was conducted for each population, which took symmetric and asymmetric components into account.

### Methods–variation in non-morphometric characters

#### Subanal fasciole, projection of the labrum, interradial structure of the paired petals, and granulation of the periplastronal area

**Fig 4:**
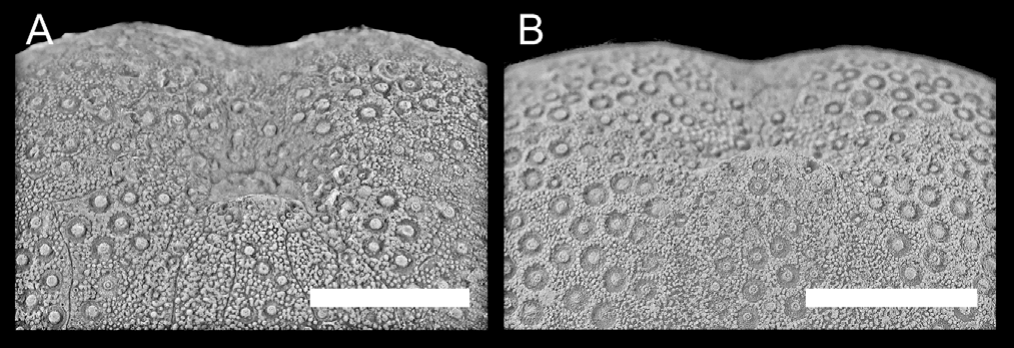
Variation in the projection of the labral plate. (A) Weakly projecting, not covering the peristomal opening (GSUB E3847, Erwitte area, Germany), (B) strongly projecting, covering completely the peristomal opening (MB.E.8251, Liencres area, Spain). Scale bars equal 1 cm.

To define the degree of coverage by the labrum and the projection of the labrum, three descriptive states were used: *i)* open peristome (Fig 4A), *ii)* peristome is completely covered (labrum reachs the frontal margin of the peristome), and *iii)* the labrum exceeds significantly the margin of the peristome (Fig 4B). In terminology for the development of the subanal fasciole, Néraudeau and colleagues [47] was followed. According to the terminology and classification of Rowe [16], Fouray [37] and Olszewska-Nejbert [24], the populations were studied to compare the development of the interradial structure of the paired petals and the granulation of the periplastronal area.

#### Pore pair numbers versus test length

The number of pore pairs from the Erwitte and the Liencres populations were counted with the help of the image analysis software ImageJ [48]. The pore number averages of the anterior and the posterior paired petals of each specimen [(R+L)/2] were plotted against the test length. An ANCOVA was computed to assess for significance of differences between slopes among the populations.

#### FA analysis of pore pair numbers in the anterior and the posterior paired petals

The counts of the pore pairs were not repeated, as they can be confidently performed without any measurement error. Grubb’s test was applied to check for outliers, as outliers otherwise could artificially inflate the results of FA. Prior to FA analyses, a size dependency of FA was tested by calculating a Spearman rank correlation of unsigned values and averaged trait size. FA, which varies with trait size, could otherwise obscure the analysis [49]. Normal distribution was assessed with a Shapiro–Wilk test, to test for ideal FA, since the property of FA is a normal distribution of right side–left side differences with a mean of zero, and hence to exclude the possible occurrence of antisymmetry [49]. A one-sample t-test was performed to test if the data sets deviate from a mean of zero, in which case DA would be present.

Following Palmer and Strobeck [49, 50], two indices were computed to estimate developmental instability. FA1 displays the mean of unsigned differences between the right and left sides [mean |R-L|], and FA4a assesses the variance within a given trait [0.798√var (R–L)]. The FA4a index is a modified version of the FA4 index, which has the advantage (compared to FA1) of not being biased by the presence of DA [7]. If DA is present, the influence of DA on the asymmetry values could be assessed by comparing DA, as the mean (R–L), with the value of FA4a. In the case of DA not exceeding FA4a, the variation in the trait is mainly due to developmental instability [50]. Levene’s test was conducted by using the unsigned values to evaluate differences of FA among populations.

## Results

### Morphometric variation

#### Shape analysis

**Fig 5:**
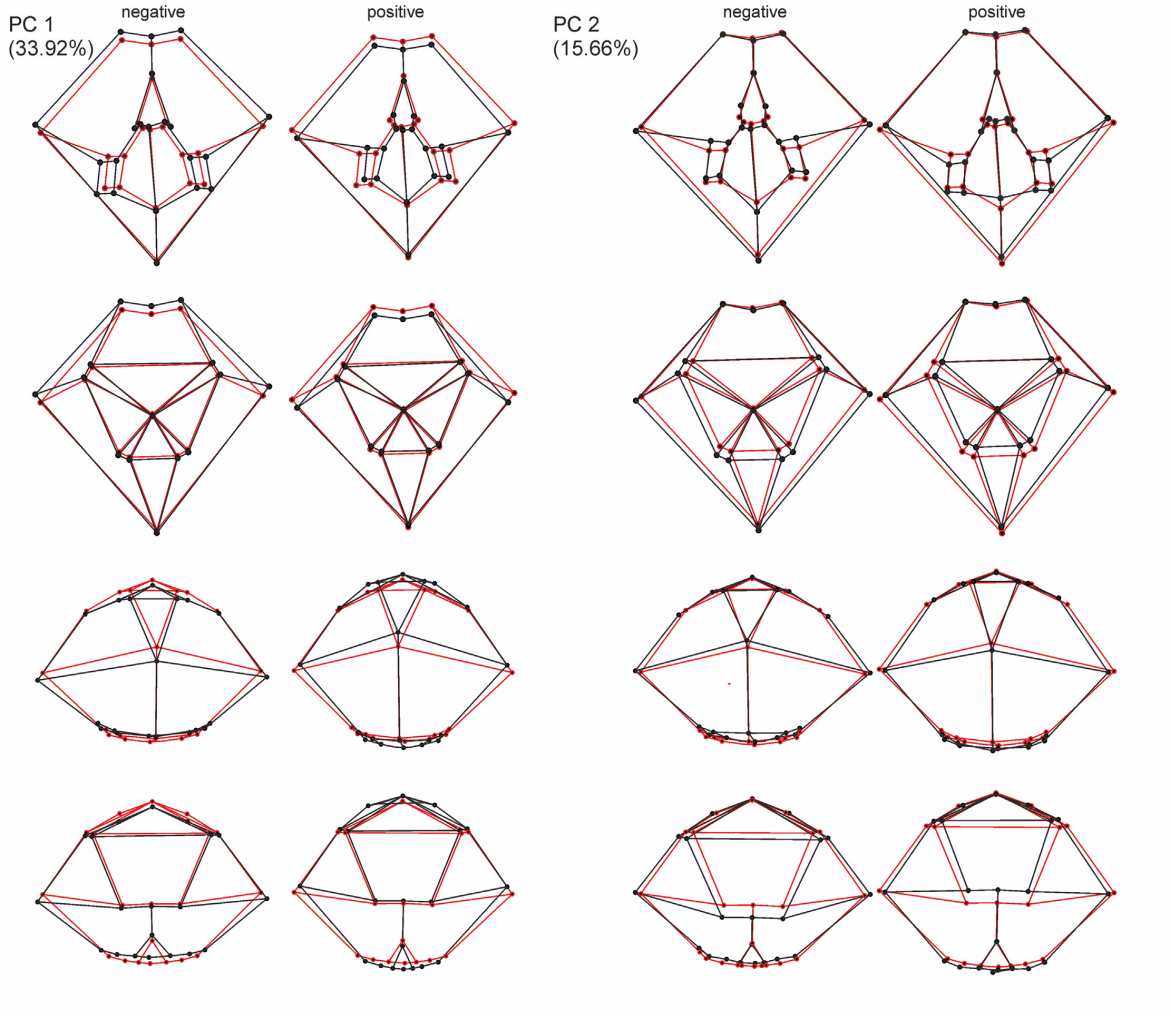
Global shape variation. Wireframe graphs show the shape changes from the mean shape (red) to shape changes associated within PC1 and PC2 with a negative and positive direction.

**Fig 6:**
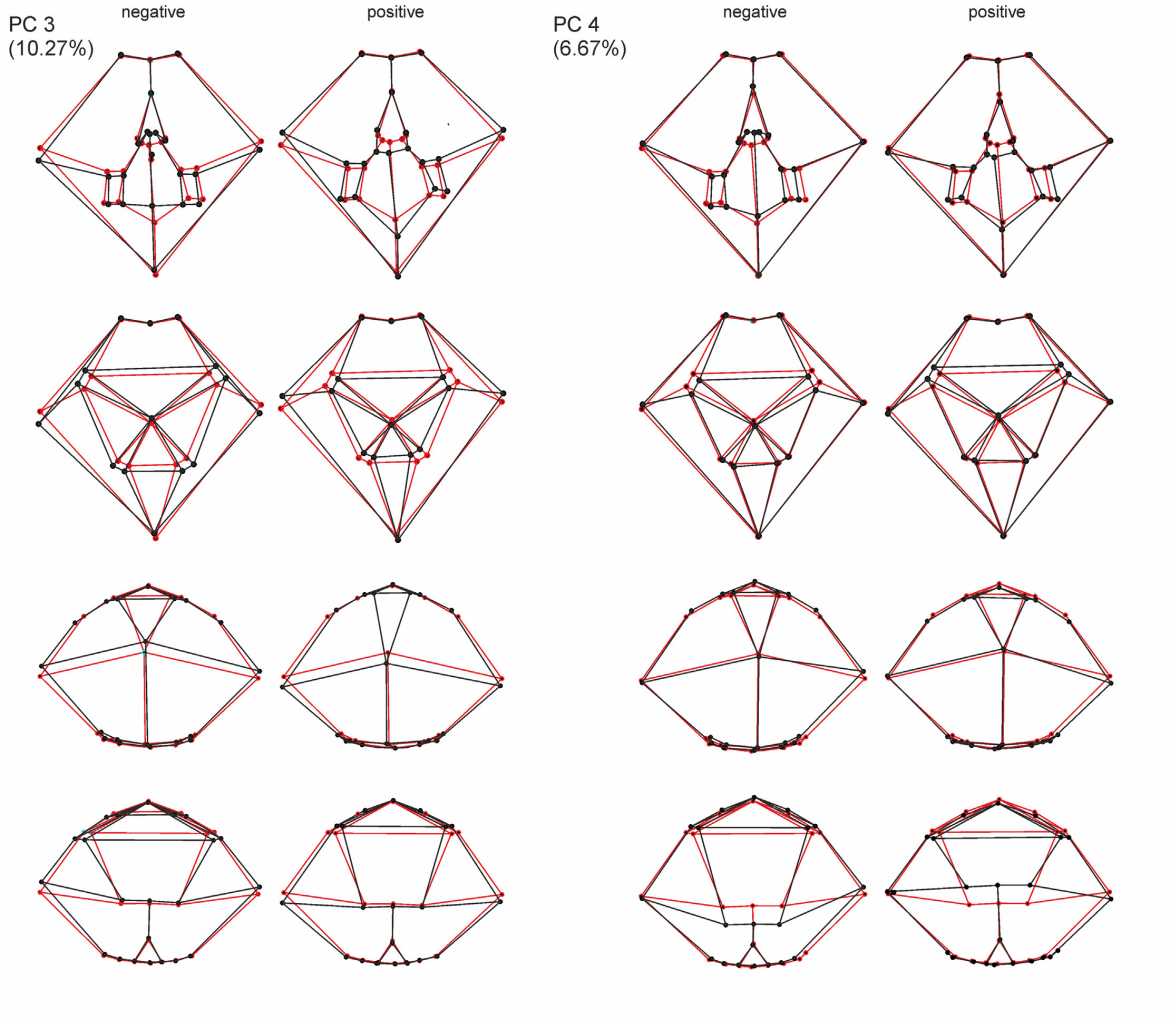
Global shape variation. Wireframe graphs show the shape changes from the mean shape (red) to shape changes associated within PC3 and PC4 with a negative and positive direction.

**Fig 7:**
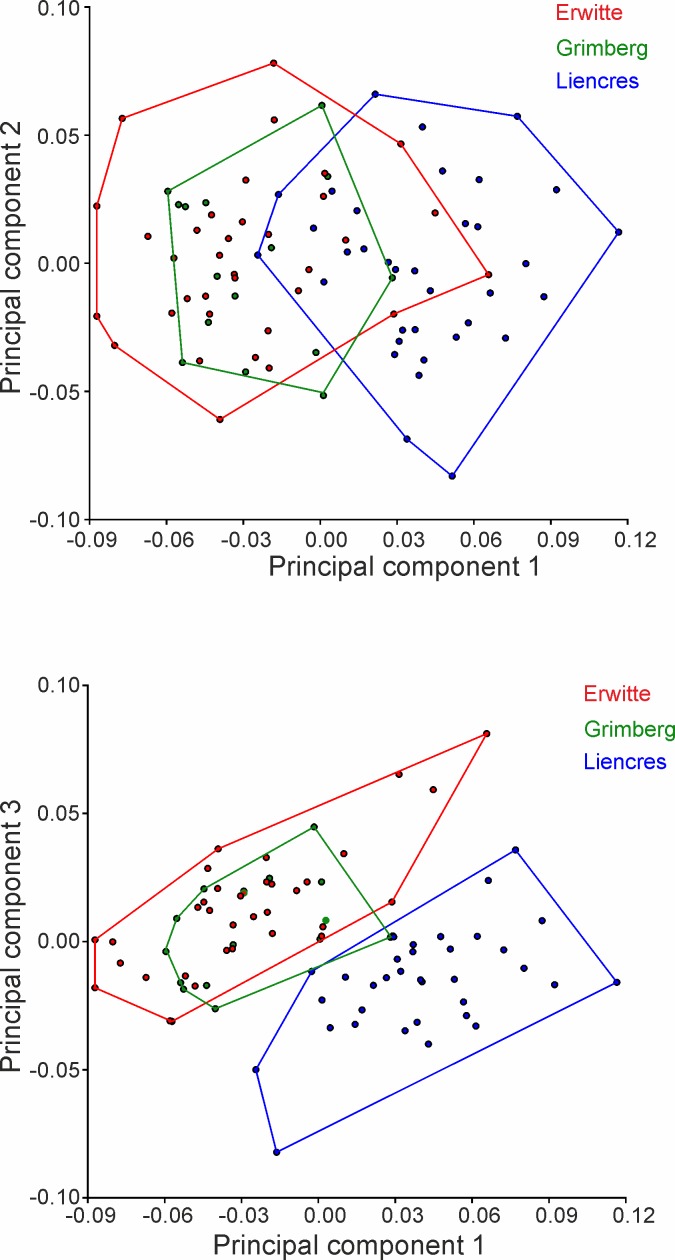
Principal component analysis scatter plots. (A) PC1 versus PC2. (B) PC1 versus PC3.

**Fig 8:**
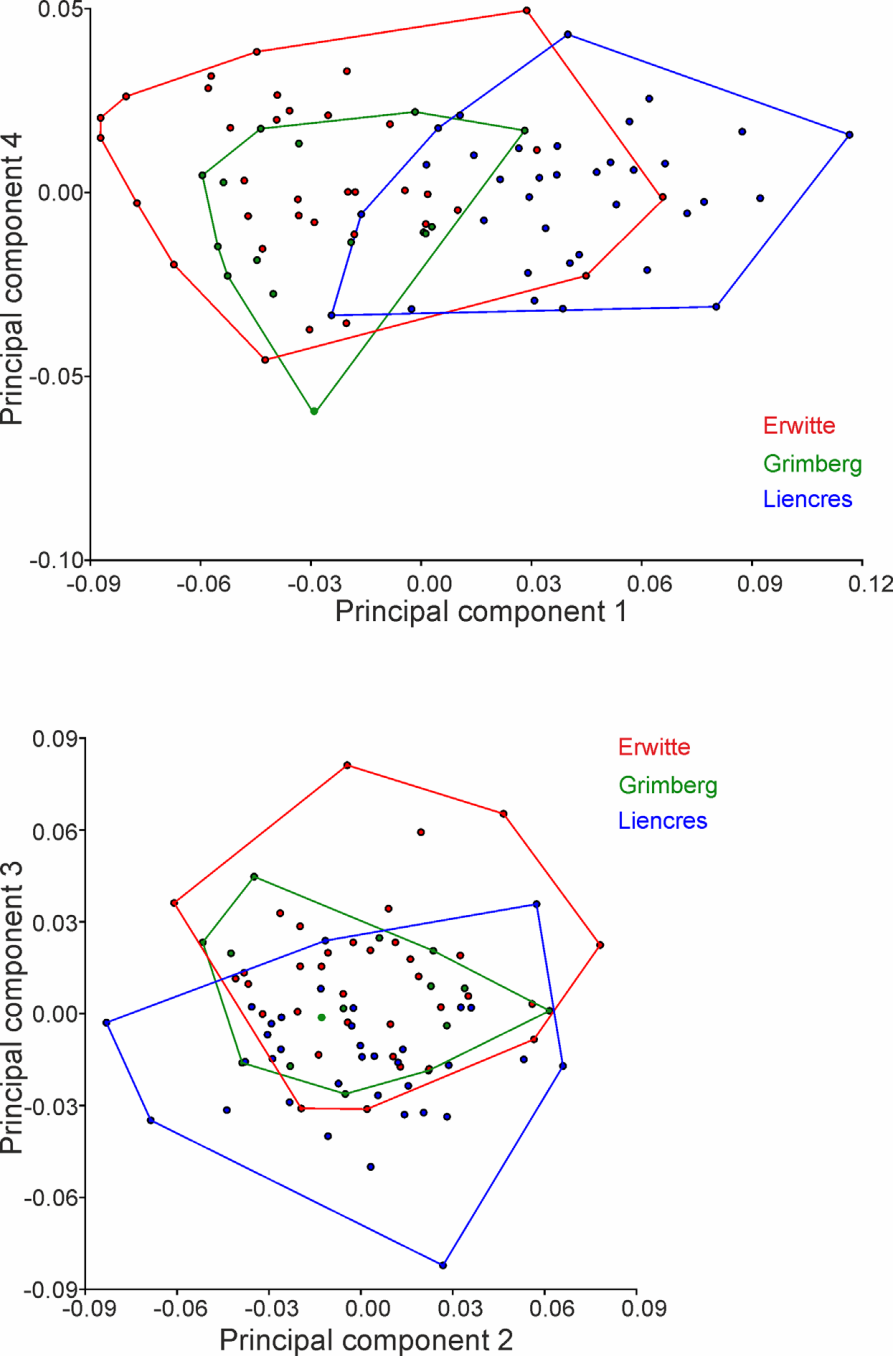
Principal component analysis scatter plots. (A) PC1 versus PC3. (B) PC2 versus PC3.

The first four PCs account for 66.53% of the total shape variance (Figs 5 and 6). PC 1 reveals the best separation between the German populations and the Spanish population (Figs 7 and 8); they reveal, however, some overlap, but a larger dispersion of the Spanish population is found along the positive direction of PC 1—both German populations disperse rather in a negative direction. The latter both have in any of the describing principal components large overlaps.

PC 1 (33.92% of total variance) is linked in a positive direction with a higher positioned periproct and ambitus, and the widest point of the test is set in the posterior position. The sternal plates are generally slimmer and shorter, the periplastronal ambulacral plates are set more in the anterior direction, and the plastronal area is more inflated. Furthermore, the shape of the test is more tumid, less elongated, and taller with a strongly positive PC 1.

PC 2 (15.66% of total variance) is associated in a positive direction with a posterior shift of the periplastronal ambulacral plates, combined with shorter sternal plates. The paired petals are less extended.

PC 3 (10.27% of total variance) pertains to shape variations with a change in a positive direction in a lower-levelled ambitus and periproct. Furthermore, the widest point is more anteriorly situated, and the more asymmetric sternal plates are found in a rather anterior position and have a broader contact to the labrum. The periplastronal ambulacral plates moved in the anterior direction, and the paired petals are shorter by a concurrent shift of the apical shield closer to the anterior margin than in relation to a negative direction.

In the positive direction of PC 4 (6.67% of total variance) a change in shape is illustrated mainly by a lower test with a higher position of the deepest impression in the notch, and the maximum width is found in the posterior direction. The apical shield is closer to the anterior notch, combined with longer anterior paired petals. The sternal plates are more asymmetric than in a change in the negative direction, and are in a broader contact to the labral plates; the periplastronal ambulacral plates are positioned in the anterior direction.

#### Fluctuating asymmetry analysis

**Fig 9:**
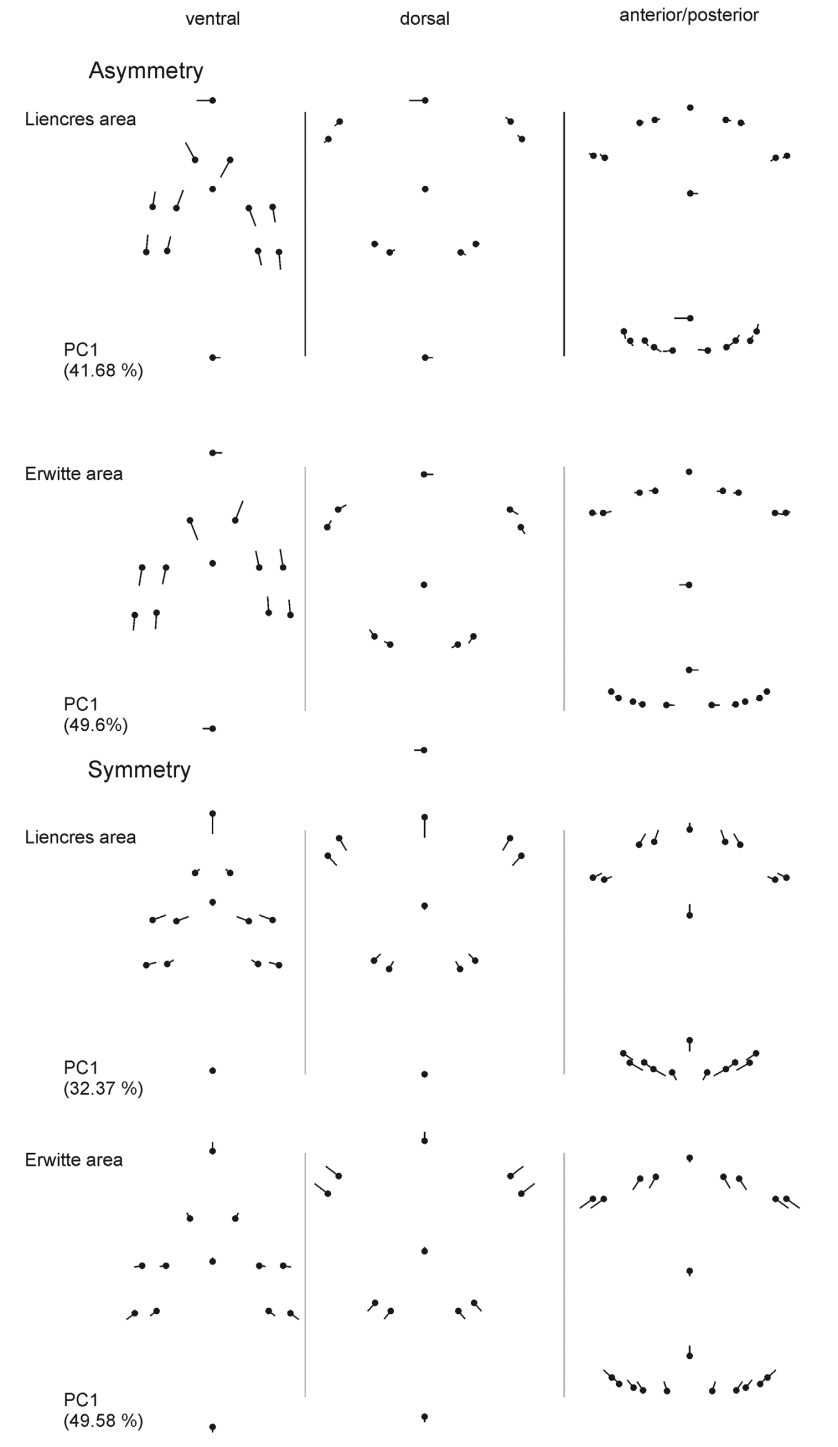
Shape variation of the asymmetric and symmetric component. Shape variation on PC1 for the population from the Liencres and the Erwitte area.

Directional asymmetry is statistically significant in the Erwitte population for the global shape variation and for the separately analysed periplastronal ambulacrals and paired petals; in the Liencres population, DA is significant in the paired petals, in the global shape variations, and in the periplastronal ambulacrals it is significant only at a *P*-value level of 0.05. FA is significant for all analysed shape variations in the Erwitte and in the Liencres populations (Table 2). The measurement error is compared to the interaction term (individual-by-side) is only minor throughout and is therefore negligible. In both populations, FA is most conspicuous in the oral ambulacral plates and shape changes refer mainly to shifts of the periplastronal ambulacral plates along the posterior–anterior axis (Fig 9, see S2–S4 Figs for PC2, PC3, PC4 respectively), whereas variations in the symmetric component are largely associated with the shape of the plastron. Differences in the amount of FA among both populations (see S1 Table for individual FA scores) were for all shape variations not significant, as revealed by Levene’s test (global shape: *P* = 0.49; periplastronal ambulacrals: *P* = 0.87, paired petals: *P* = 0.37).

**Table 2:** Procrustes ANOVA results for the populations from Erwitte and Liencres area.

population	trait	Effect	SS	MS	df	F	*P* (param.)
Erwitte area							
	general shape	Individual	0.26086047	0.00054346	480	6.76	<0.0001
		Side	0.0053173	0.00037981	14	4.72	<0.0001
		Individual x Side	0.03602375	8.041E-05	448	38.12	<0.0001
		Measurement error	0.00201864	2.1093E-06	957		
	periplastronal ambulacralia	Individual	0.28937101	0.00100476	288	3.86	<0.0001
		Side	0.01070259	0.00133782	8	5.14	<0.0001
		Individual x Side	0.06665542	0.00026037	256	68.21	<0.0001
		Measurement error	0.00214141	3.8171E-06	561		
	paired petals	Individual	0.18856263	0.0009821	192	10.21	<0.0001
		Side	0.0028151	0.00056302	5	5.85	<0.0001
		Individual x Side	0.01538751	9.6172E-05	160	16.46	<0.0001
		Measurement error	0.00212048	5.8416E-06	363		
Liencres area							
	general shape	Individual	0.21060018	0.00041294	510	5.68	<0.0001
		Side	0.00283036	0.00020217	14	2.78	0.0005
		Individual x Side	0.03462216	7.2736E-05	476	36.89	<0.0001
		Measurement error	0.00200114	1.9716E-06	1015		
	periplastronal ambulacralia	Individual	0.26783721	0.00087529	306	3.52	<0.0001
		Side	0.00539528	0.00067441	8	2.71	0.0069
		Individual x Side	0.06765032	0.00024871	272	60.98	<0.0001
		Measurement error	0.00242686	4.0788E-06	595	-0.01	
	paired petals	Individual	0.1466068	0.00071866	204	7.31	<0.0001
		Side	0.00283686	0.00056737	5	5.77	<0.0001
		Individual x Side	0.01671819	9.8342E-05	170	17.41	<0.0001
		Measurement error	0.00217429	5.6475E-06	385		

### Variation in non-morphometric characters

#### Subanal fasciole, projection of the labrum, interradial structure of the paired petals, and granulation of the periplastronal area

**Fig 10:**
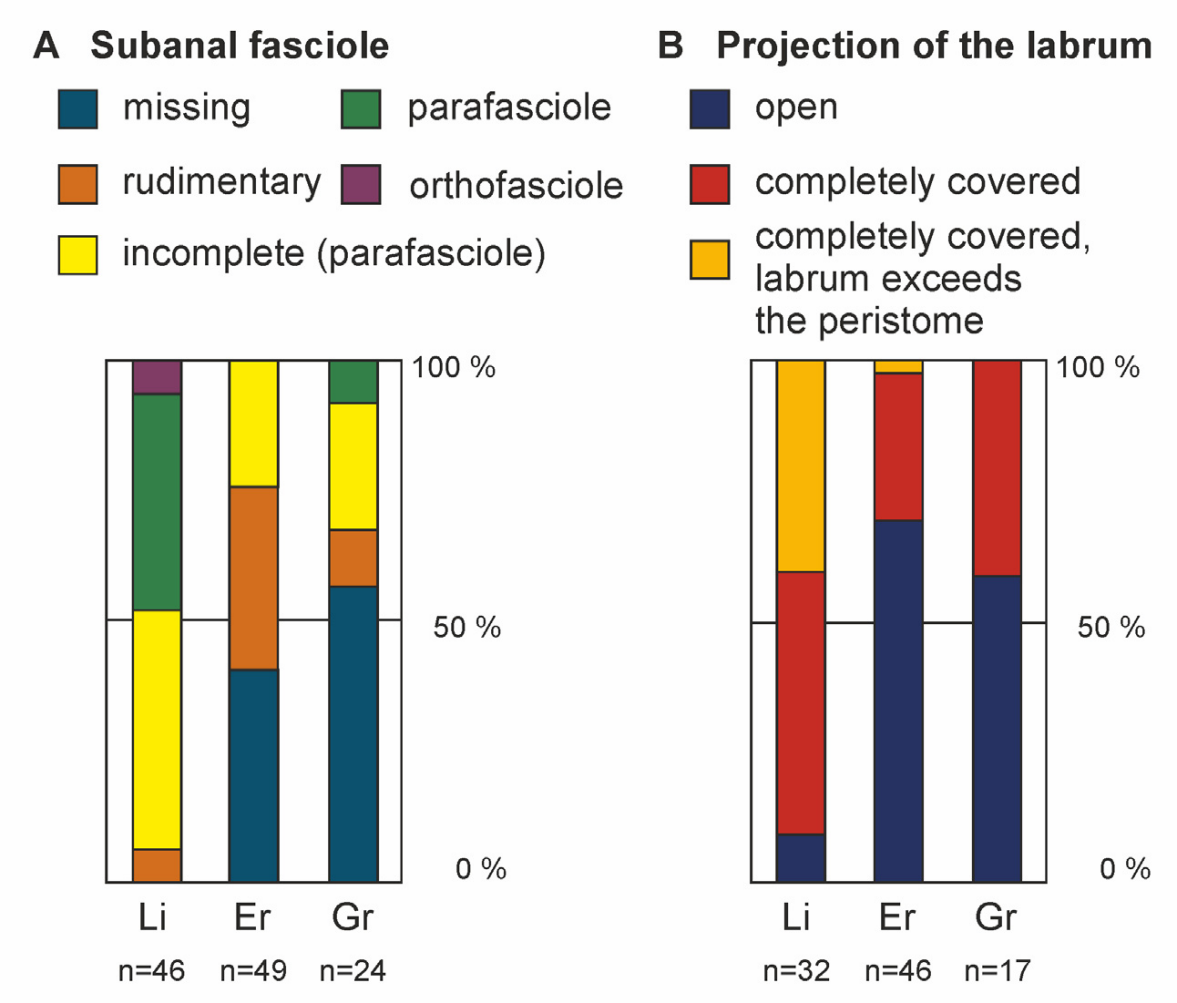
Variation in development of the subanal fasciole. Bar charts indicating the percentage for the particular populations in (A) the development of the subanal fascioles, (B) the development of the projection of the labrum (Li = Liencres area; Er = Erwitte area; Gr = Grimberg).

**Fig 11:**
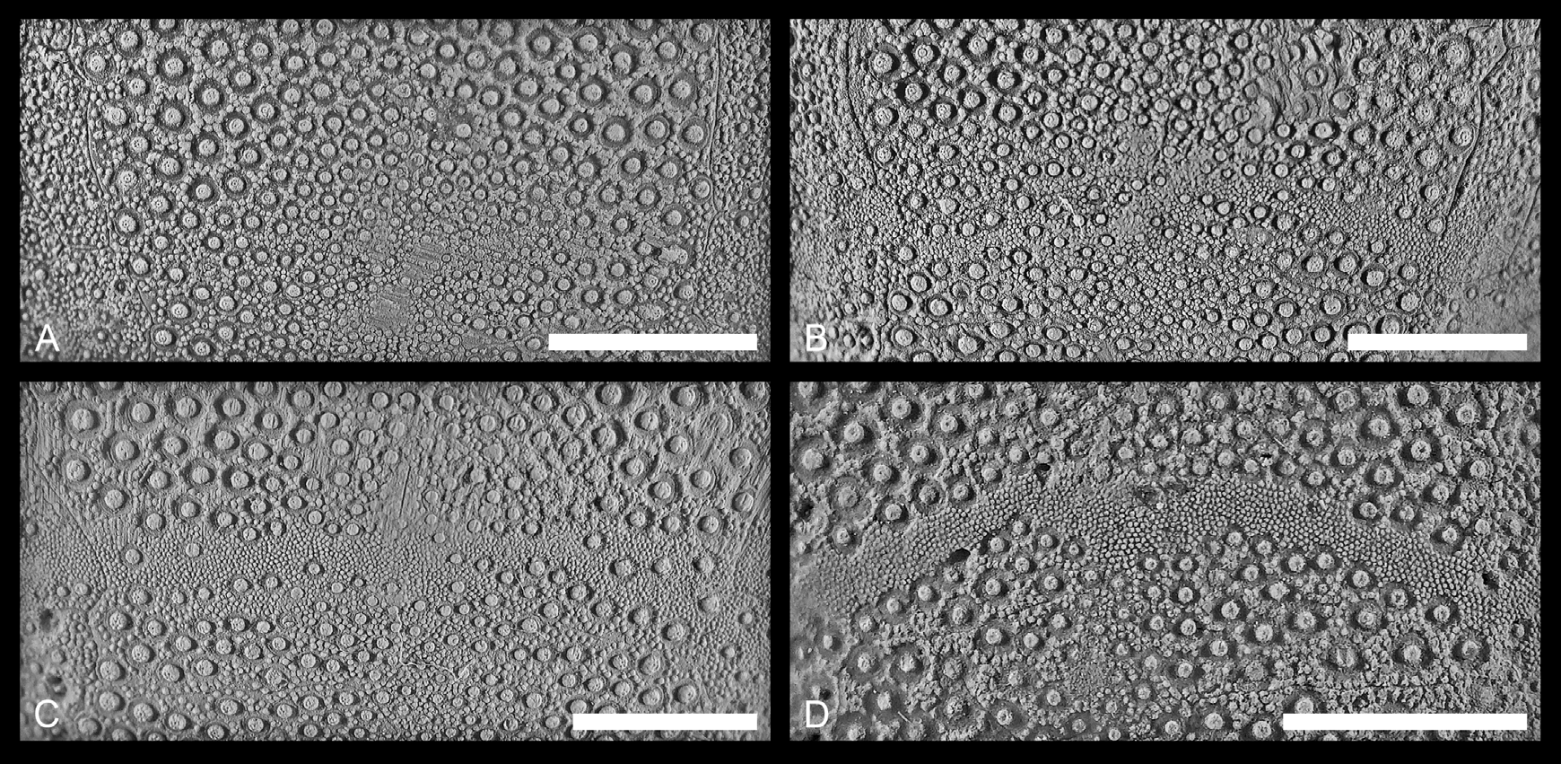
Photographs illustrating variation in development of the subanal fasciole. (A) Not present (GSUB E3847, Erwitte area, Germany). (B) Incomplete (GSUB E3850, Erwitte area, Germany). (C) Protofasciole (BGR X 06195, Grimberg IV shaft, Germany). (D) Orthofasciole (MB.E.3873, Liencres area, Spain). Scale bars equal 0.5 cm.

An obvious trend is seen in the development of the subanal fasciole, which is most pronounced in the Liencres population (Fig 10A, S2 Table), where a trace of a subanal fasciole is always present. In a large proportion of the Grimberg and Erwitte populations, on the other hand, no subanal fasciole could be detected (46% and 41% respectively, Figs 10A and 11A). Likewise, more complete types of fasciole development are found in specimens from the Liencres area (parafasciole: 41%, Fig 11C; orthofasciole: 7%, Fig 11D, S3 Table). In both populations, specimens having a parafasciole were only found in the Grimberg material; orthofascioles are completely missing.

**Fig 12:**
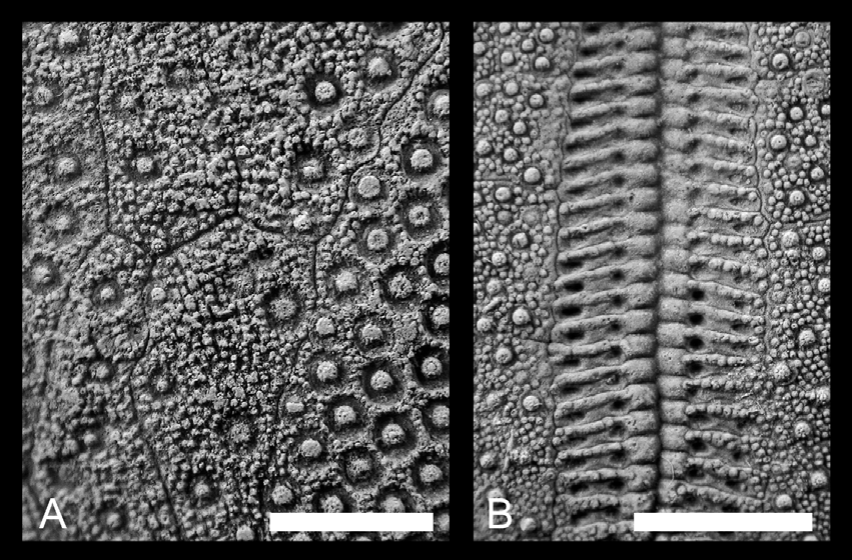
Development in the structure of periplastronal area and the interporiferous area of the paired petals. (A) Granular periplastronal area (MB.E.8196, Liencres, Spain). (B) Subdivided interporiferous area of the paired petals (GSUB E3867, Erwitte area, Germany). Scale bars equal 0.5 cm.

A similar tendency is found for the projection of the labrum. A minor part of the Liencres material has an uncovered peristome. Predominantly, the labrum is covering and/or even exceeding the margin of the peristome. This is in contradiction to the observations made in the German populations, in which the majority of the specimens have only weakly projecting labral plates; the peristomes are, to a large extent, uncovered. No significant differences between all populations were observed in the development interradial structure of the paired petals (Fig 12) and the granulation of the periplastronal area (results are not shown).

#### Pore numbers versus test length

**Fig 13:**
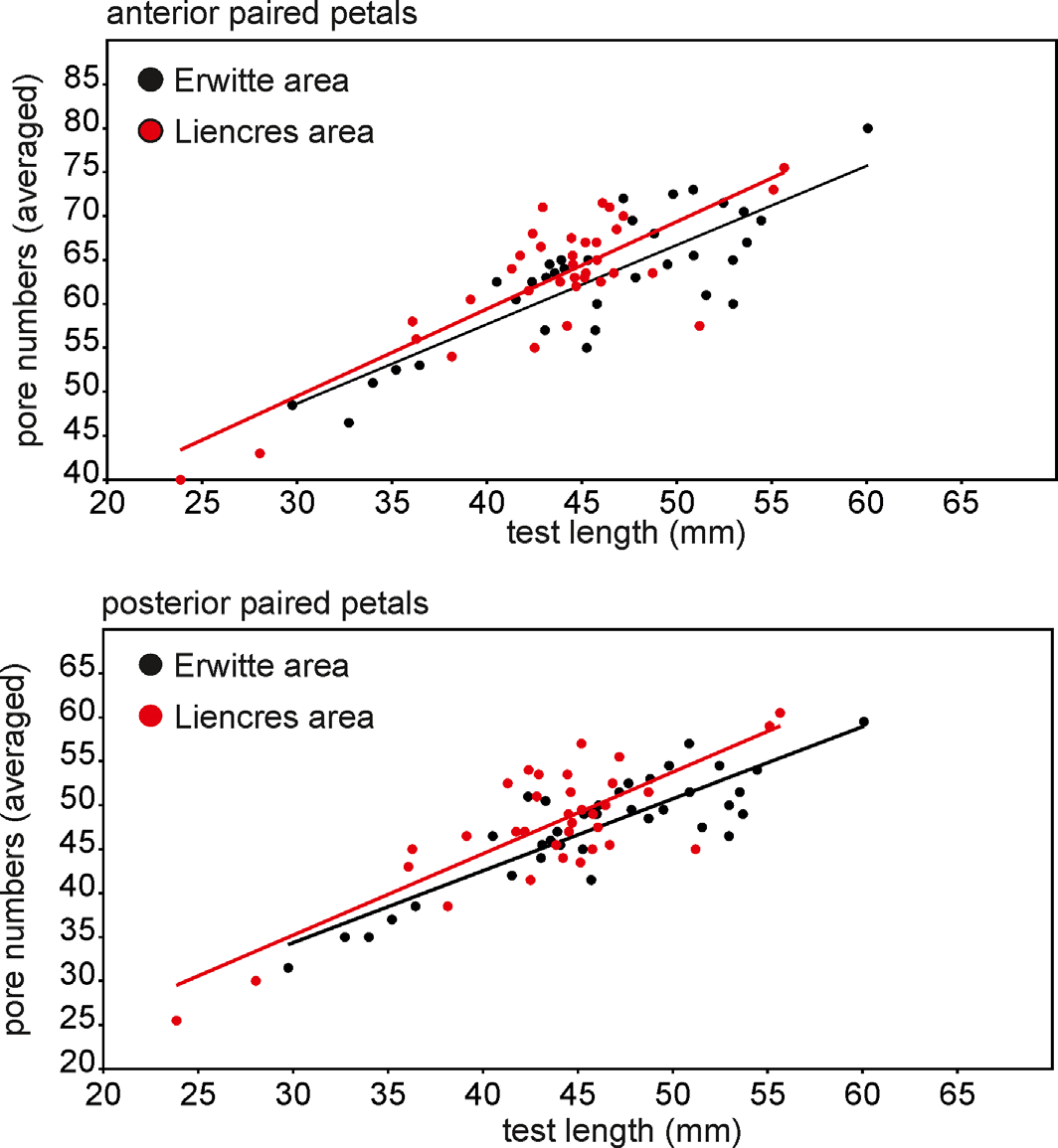
Bivariate scatter plot of the numbers of pore pairs against test length.

The slope for the anterior paired petals of the Liencres population differs somewhat from the Erwitte population (0.93 and 0.90 respectively, Fig 13). The ANCOVA, however, revealed no significant differences among both populations (*P* = 0.57). Similar results are found for the posterior paired petals, and the slope in the Liencres population is larger (0.93) than in the Erwitte population (0.82), but insignificantly different (*P* = 0.45).

#### Fluctuating asymmetry analysis for the pore numbers in the paired petals

The values of each FA index and mean asymmetries are given in Table 3 (see S4 Table for individual FA values). One outlier from the set of posterior paired petals was excluded from the following analyses according to Grubb's test. All studied traits reveal a normal distribution (*P*>0.05); accordingly, antisymmetry is not present in the studied traits.

**Table 3:** Results of the FA analysis considering the pore pair numbers.

	FA1	FA4a	mean (R-L)	t-test (*P*-values)
Liencres				
anterior petals	2.78	2.26	1.89	0.00031
posterior petals	1.75	1.63	-0.26	0.46
Erwitte				
anterior petals	1.2	1.16	0.57	0.026
posterior petals	1.57	1.6	-0.46	0.1863

**Fig 14:**
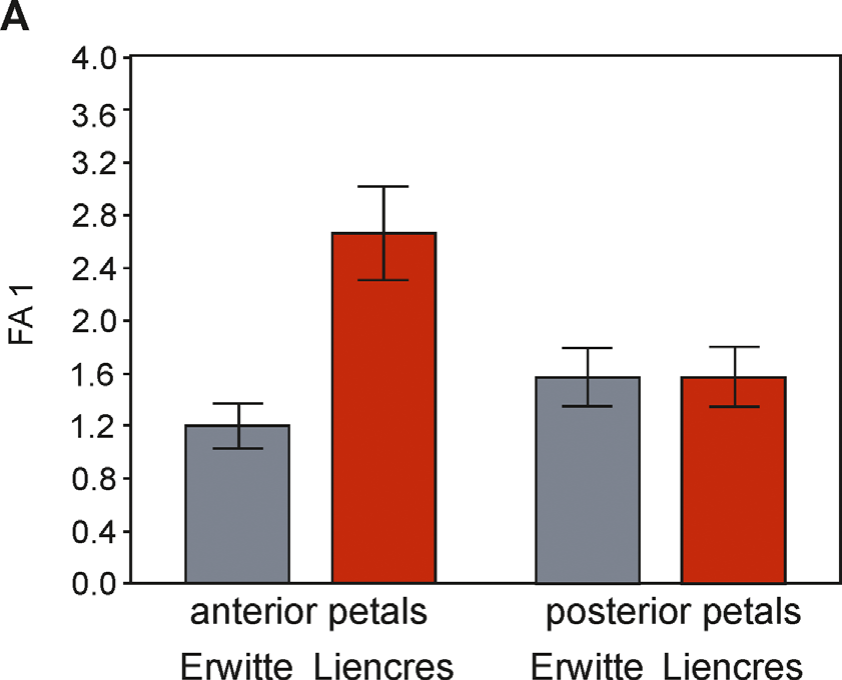
FA1 scores of the pore pairs numbers. Bar charts of the FA scores (FA1 index) for pore counts of the anterior and the posterior paired petals of the Liencres and the Erwitte population.

A correlation of FA with the trait size could neither be detected in the anterior nor in the posterior paired petals. The differences of FA between the Erwitte and Liencres populations in the posterior petals are not significant, as concluded by Levene’s test (*P* = 0.84); DA could not be detected. The presence of DA in the anterior petals was confirmed by a t-test in both samples. Estimated by a comparison of the mean (R–L) and FA4a for each sample, the values of the FA4a are larger than the mean (R–L) in both cases; accordingly, the asymmetry variation accounts largely for FA. The FA scores (FA1 & FA4a) for the anterior petals in the Liencres population are significantly higher (*P*<0.001) than in the Erwitte population (Table 3 and Fig 14). The range in between-sides differences in this trait is sufficient for avoiding biased estimates of FA, as suggested by Swain [51] for the reliability of meristic traits to assess developmental instability.

## Discussion

### Shape variation

The Spanish and the German populations of *M*. *brevis* are distinguished by the displacement of the periproct and the ambitus, which are generally in a higher position in the Spanish specimens. Furthermore, the shape of the plastron is slimmer, shorter and more inflated in the Liencres population than in the German populations. The latter populations show a large overlap in their morphospace and are virtually indistinguishable. The morphological differences between the German and the Liencres populations argue for an adaption to the grain size of their host sediments. With regard to the burrowing behaviour, the Spanish and the German populations must have been different. An inflated plastron, like in the Liencres populations, is found in deeper burrowing spatangoids [35]. The position of the periproct is related to the burrowing depth of the animal, and deeper burrowing species have a higher positioned periproct than shallow or epifaunal burrowers [24, 33]. The generally higher situated periproct in the Liencres sample supports the interpretation of a deeper burrowing behaviour.

A similar pattern for the variation in the plastron shape in relation to the sediment was found in the extant spatangoid *Echinocardium cordatum* among populations from the waters of Great Britain and New Zealand [31]. Curiously, however, the relation to the grain size is in contradiction to the pattern which can be observed in *M*. *brevis*. In *E*. *cordatum*, however, broader shapes are related to mud and clay, and narrow shapes to sand and gravel. Only the degree in inflation of the plastronal area is in both species linked to coarser substrates. These contradictory outcomes are probably related to differences in locomotion and burrowing mechanisms among both species, which demonstrates interspecific variations [33, 52]. *Echinocardium cordatum*, for example, is a deeply burrowing species [33], which is in contrast with the assumed shallow burrowing depth of *M*. *brevis*. These different patterns in plastron shape are, however, worth to be studied in more detail elsewhere. Regardless of these deviations, both studies show a strong linkage between the substrate and plastron shape. Egea and colleagues [53] mention that phenotypic plasticity has an influence on the morphology of *E*. *cordatum*, unfortunately without giving specific examples. Another example of how the morphology in spatangoid echinoids changed in relation to the sediment is given by the fossil *Toxaster granosus kiliani* (Lower Cretaceous) [34]. It responded to an increase in the abundance of clasts in the sediment by an inflation of the test and by a higher positioned periproct. This pattern was also observed in *E*. *cordatum* from northern France [34]. These observations correspond to the results found in *M*. *brevis*.

The phenotypical differences within *M*. *brevis* either relied on genetic differentiation or were reasoned in phenotypic plasticity. Although phenotypic plasticity is ubiquitous in organisms, it is challenging to draw conclusions about phenotypic plasticity in the fossil record (discussed for the case in ammonoids [54]). McNamara and McKinney [55], Chauffe and Nichols [56], and West-Eberhard [3], however, proposed criteria to evaluate phenotypic plasticity in the fossil record. It was argued that phenotypic plasticity can be assumed, for instance, if the modified trait is not occurring uniquely; all forms of (isogenic) taxa would modify in the same way, and other related taxa would modify in a similar way (if being exposed to the same stimuli). This evaluation can be hampered by the fact that phenotypic plasticity, however, is a property of a genotype. Accordingly, the reaction norm (the range of phenotypic expression of a genotype across an environmental variable) can vary within or between populations [57]. In addition, initially, plastic traits can change by genetic accommodation, hence being fixed [3, 58, 59]. Nevertheless, the relationship between the position of the ambitus, the periproct respectively, and the grain size in fossils *T*. *granosus kiliani*, *M*. *brevis* and the extant *E*. *cordatum*, as mentioned above, suggests an influence of phenotypic plasticity. This is possibly true for the development of the plastron shape as well, but further confirmation is needed.

The populations of *E*. *cordatum* from New Zealand and Great Britain, analysed by Higgins [31] with respect to the plastron shape, are yet genetically divergent [53], but whether these genetic differentiations contributed to the shape variations is unclear. The high and rapid dispersal potential in *Micraster*, due to their planktotrophic larvae [60], would have enabled gene flow between the Spanish and German populations. However, gene flow between both areas may have been asymmetric; several examples suggest that a north to south directed migration of taxa existed during the Late Cretaceous [61]. In the case of the cryptic species complex *E*. *cordatum* [53, 62], planktotrophic larvae allowed for widespread settlement of genetically homogeneous clades. However, the possibility of genetic variation in *M*. *brevis* cannot be totally excluded. Other shape variations are shared by all three populations and cannot be referred to distinct habitats. Thus, insofar as it is not possible at present to associate these variations with an environmental gradient, their development may be attributed to genetically influenced variation.

### Fluctuating asymmetry

Environmental stressors, like assumable temperature clines, potentially could have contributed only to a negligible amount of FA. Moreover, shared inheritable factors (e.g. effects of non-additive gene–gene interactions, see [63]) could have contributed to similar patterns and degrees of FA in the periplastronal ambulacrals found in both populations. This is, of course, very speculative and needs to be discussed further by inclusion of more populations of different habitats. Additionally, assessments on phenotypic variance can be biased by time averaging, which potentially inflates phenotypic variance within larger time intervals [64]. However, it was tried to limit this bias by restricting the time interval under study as much as possible.

**Fig 15:**
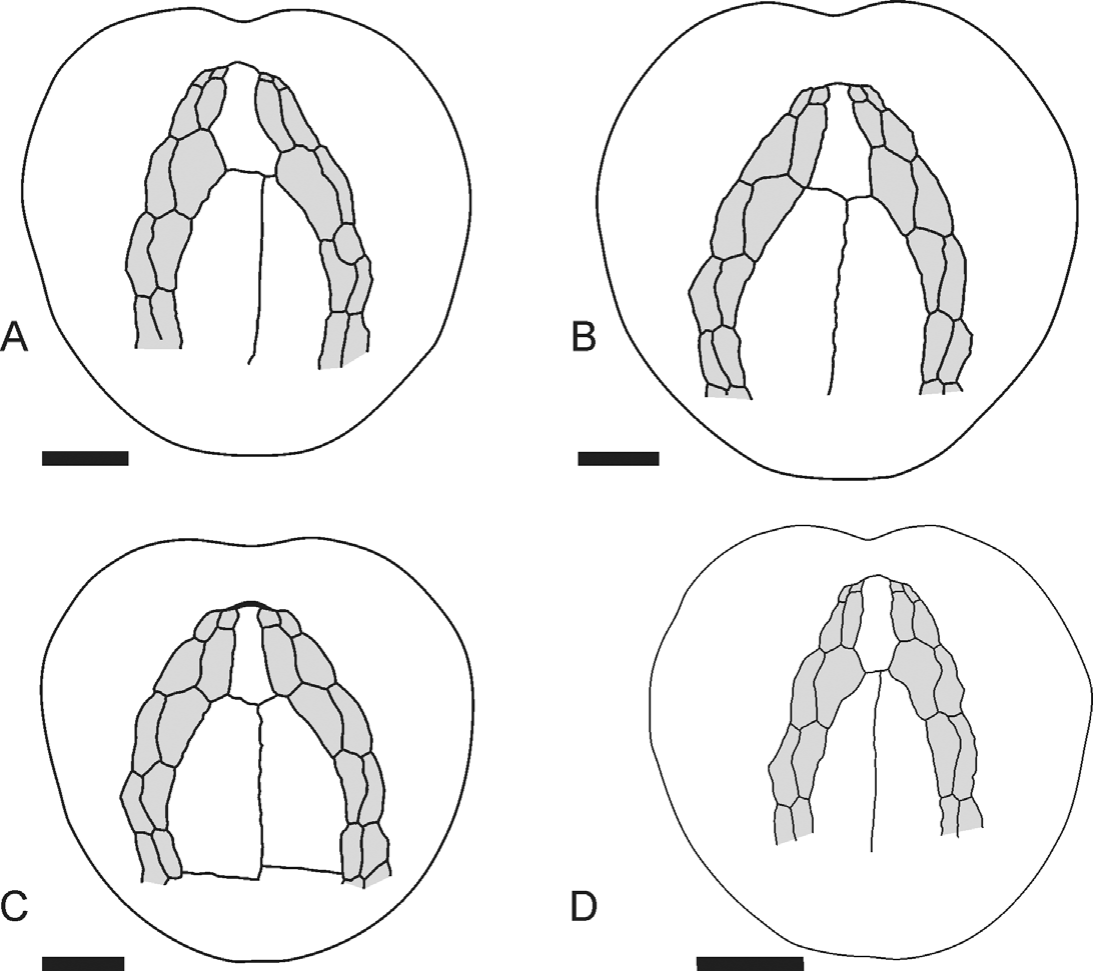
Sketches illustrating the variation in the periplastronal ambulacral plates. (A-B) Conspicuous asymmetric development. (C-D) Symmetric development, with longer ambulacral plates (C) and shorter plates (D) next to the labrum (A: MB.E.8196, Liencres area, Spain; B: GSUB E3847, Erwitte area, Germany; C: GSUB E3867, Erwitte area, Germany; D: MB.E.8257, Liencres area, Spain). Scale bars equal 1 cm.

Remarkably, variation within individuals can exhibit conspicuous variability in the periplastronal ambulacral plates, in which one plate can be very shortened, while the opposing plate can show a very extended shape (Fig 15). These within-individual variations reflect intra-populational variation in the development of these plates, as described by the first four principal component factors for global shape variations. The pattern of the symmetric shape and the asymmetric shape variation, however, are not congruent. This disagreement is possibly explained by the fact that a large amount of variation is attributed to the shape variation in the plastron, which is associated with the position of the periplastronal ambulacral plates.

These stochastic variations might give some clues to the developmental process of this trait. Inferred from the probability that these extreme asymmetries are a result of stochastic gene expression, i.e. fluctuations in the amount of a gene product [65], it suggests that the (symmetric) variation in these plates was a result of homometry (changes in the amount of gene products, see [66]). McNamara [67], however, suggested that displacement in the periplastronal ambulacral plates of spatangoid echinoids is reasoned in allometry. Studies on allometric variation in this trait of *M*. *brevis* are needed to give better ideas on their development.

A study on FA of two populations of *Echinocardium flavescens* [68] has shown that variations in the periplastronal ambulacrals are most intense along the main growth (longitudinal) axis and are, to some extent, constrained to this direction. This suggests that shape variations of FA in the periplastronal ambulacrals are, to some degree, under common developmental constraints. The amount of FA in *E*. *flavescens*, however, generally increased along the anterior–posterior axis, unlike in *M*. *brevis*, where the anterior landmarks are more influenced by FA. This assumes that regulatory mechanisms between *M*. *brevis* and *E*. *flavescens* are partially different. DA is thought to be ubiquitous in the skeleton of irregular echinoids, which is assumed to be related to the asymmetry of the digestive system [69, 70]. Accordingly, the occurrence of DA in at least one sample (Erwitte population) is not surprising.

### Variation in non-morphometric characters

#### Subanal fasciole and peristome coverage by the labrum

The subanal fasciole is better developed in the Liencres specimens, while it is often totally absent in the German populations. The task of the subanal fasciole is to provide a water current, by movement of the ciliated spines, and thus to sweep the faeces away from the body [23]. A sustainment of such a feature is more reasonable at a deeper burrowing depth. The development of the fasciole had been linked to the nature of the sediment in several taxa of fossil spatangoids by previous authors [32, 47], which is in agreement with the present observations on *M*. *brevis*. Néraudeau [71] already stated that the occurrence and development of fascioles in general are influenced by phenotypic plasticity. Fascioles (e.g. subanal, or lateral fascioles) in fossil spatangoids are better developed if these inhabited finer grained sediments, whereas in coarse grained sediments fascioles tend to be lesser developed, or even become lost. As this pattern is in an inverse relation to the here recognised variation, it has to be considered that the need for fascioles is influenced by the burrowing behaviour [23], fascioles are generally rather found in deeper burrowing taxa. Accordingly, a need for fascioles corresponds to the permeability of the sediments (governed by grain size) and burrowing depth. According to the tendency for better-developed subanal fascioles in the Liencres population, it is probably related to the foregoing burrowing depth, due to coarser-grained sediment.

It is unclear to which function the projection of the labrum is related [72]. Nichols [36] interpreted this development as being associated with a change in feeding habits, from a food supply from underneath the peristome towards a transport of particles from the surface to the peristome via the frontal notch. However, so far there is no clear evidence in support of this idea. A stronger projection of the labrum can be observed within the evolution from *M*. *leskei* (late Turonian) to *M*. *coranguinum* (late Coniacian), e.g. [23]. *Micraster brevis* is assumed to be a side branch of this lineage [17, 18]; thus, it is remarkable that a change of the labrum projection apparently took place independently in different species of *Micraster*. Accordingly, the development of the labrum in *Micraster* would then be homoplastic, as a result of either parallelism or convergence. An increase in the projection of the labrum also appeared in other spatangoid lineages, e.g. [73], which supports the idea of homoplastic development in this trait. Moreover, the here-observed differences in the degree of projection in the labrum between the populations argue for a large influence of the environment on the development of the labrum. To what degree this development was mediated through phenotypic plasticity or genetically determined must remain unclear without comparable case studies.

Interestingly, the interradial structure of the paired petals and the granulation of the periplastronal area are the most invariant traits considered here. This finding could indicate low genetic variation, and/or low environmental influences, or high developmental stability, or canalization, which buffers against any perturbations.

#### Pore numbers versus test length

Elongated pores such as those in the paired petals of *Micraster* are linked to ambulacral tube feet, which are specialised for gaseous exchange [38]. Accordingly, as suggested by the works of Stokes [15] and Zaghbib-Turki [32], an adaptation towards higher temperatures in southern palaeolatitudes, by an increase in the petaloid pore pair numbers, could have been expected in the population from the Liencres area. However, no significant differences between the populations could be found. This could argue either for insufficient statistical power or for similar temperatures in both realms. Unfortunately, reconstructions of palaeotemperatures of the early Coniacian of these areas are not available.

#### Fluctuating asymmetry analysis for the pore pair numbers in the paired petals

The pore numbers in the anterior paired petals show a higher amount of FA in the Liencres population, which indicates a higher level of developmental instability, caused by either environmental factors or genetic stressors. For instance, possible higher sea temperatures could have posed an environmental stressor in this case, as water temperatures and oxygen content are intimately linked, and must have served as a selective force for this trait. It is suggested [74, 75] that functionally important traits are more stable in development and, thus, reveal only low levels of FA, as they would be subjected more to stabilising selection. As the pore pairs in the paired petals have a highly important function, however, it could therefore be assumed that the Liencres population was exposed to non-stabilising selection.

Metric FA analysis of the ambulacral plates, including the paired petals, revealed no significant differences among both populations. This is in contradiction to the results of the meristic analysis, which suggests either insufficient sample sizes to detect significant differences in FA of shape variation, or that different processes in development of the shape and the pore pair numbers were involved. The latter idea is supported by comparisons of individual metric FA scores (computed only for the anterior paired petals) with the meristic unsigned asymmetry values of the paired petals, in which values of either analysis are only weakly to moderately related (see S5 Fig). However, comparisons of FA analysis considering the position and the numerical values of the same trait had different outcomes, in which positional FA was more sensitive to developmental instability [76], which is inconsistent with findings of the current study.

## Conclusions

This study confirms the assumption that *Micraster* developed local ecophenotypism [22]. It is most likely that especially the nature of the sediment (e.g. grain size) had a large influence on the morphology of *M*. *brevis*. The phenotypic variations suggest different burrowing behaviours of the populations in their respective environment. The morphological features in the Liencres population indicate a greater burrowing depth than in the German populations, which likely was attributed to the coarser sediment in Liencres.

Morphological variations in the position of the ambitus, the periproct and the subanal fasciole were most likely influenced by phenotypic plasticity, and potentially also the shape of the plastron. The projection of the labrum was achieved independently in different species of *Micraster*. The findings of more pronounced projecting labral plates in the Spanish sample, however, raise the question to which degree the environment played a shaping force and what kind of factors were involved. Further comparisons to distinct *Micraster* populations/species are required to gain more insights into the dependence between environmental factors and the development of this trait.

Variations due to developmental instability exist in the Liencres and in the Erwitte populations. Differences in the amount of FA, however, were only significant for the numbers of pore pairs in the anterior paired petals. Differences in environmental factors, which could have provoked these higher FA values, are unclear. Temperature gradients, for instance, to which this trait would have most reasonably responded, could not be detected, since data on palaeotemperatures are not available. Similarities among the populations in FA levels of the periplastronal ambulacral plates could have resulted from common perturbations in their developmental regulatory mechanisms.

Other shape modifications (asymmetry in the sternal plates, contact of the labrum and the sternal plates, position in the periplastronal ambulacral plates, position of the apical shield, and the widest point of the test), which were traditionally regarded as being involved in a continuous process of evolution in *Micraster*, revealed no distinct relation to specific populations and, hence, to environmental differences.

It is noteworthy that two of the here-studied traits (interradial structure of the paired petals, granulation of the periplastronal) were very robust in their development, as they reveal apparently no variation. The influence of environmental variation, however, was able to create an increase in morphological diversity, which is worth studying in the evolutionary context of the *Micraster* lineage.

Concepts and mechanisms of variability, such as phenotypic plasticity and developmental robustness, are important topics and are of great interest for evolutionary development. The majority of works addressing these concepts, however, relied on extant organisms. Data from the fossil record, however, are invaluable to our understanding of evolution in nature. Accordingly, works on these topics are worth to be extended to the fossil record and have the potential to provide important insights into trends and patterns in evolutionary history, which can be incorporated into ideas of great interest in evolutionary biology.

## Supporting Information

S1 Fig Thin sections. (A) Grimberg. (B, C) Erwitte. (D-F) Liencres. (A-C) Wackestones contain clay and silt, with relatively low content of bioclasts. (D-F) Silty packstones with abundant bioclasts and siliciclasts.
(TIF)

S2 Fig Shape variation of the asymmetric and symmetric component (PC2) for the population from the Liencres and the Erwitte area. (TIF)

S3 Fig Shape variation of the asymmetric and symmetric component (PC3) for the population from the Liencres and the Erwitte area. (TIF)

S4 Fig Shape variation of the asymmetric and symmetric component (PC4) for the population from the Liencres area and the Erwitte area. (TIF)

S5 Fig Comparison of the individual metric and meristic FA values for the specimens from Liencres area (A) and from the Erwitte area (B).
(TIF)

S1 File Material included for particular analysis. (DOC)

S1 Multimedia A 3D model of GSUB E3840 (Erwitte area) shows the landmark configuration. This file (.obj file)can be opened (import mesh) in the open-source software MeshLab (Visual Computing Lab—ISTI—CNR), available at: http://meshlab.sourceforge.net/. The landmark coordinates (GSUB E3840_picked_points.pp) can be loaded via the PickPoints function.
(ZIP)

S1 Table Individual FA scores for the metric analysis. (XLS)

S2 Table Variation in the projection of the labrum for each population. (XLS)

S3 Table Variation in the development of the subanal fasciole for each population. (XLS)

S4 Table Individual values for the pore pair numbers. (XLS)

## References

[1] Pigliucci M. Developmental phenotypic plasticity: where internal programming meets the external environment. Curr Opin Plant Biol. 1998;1: 87–91. 10.1016/S1369-5266(98)80133-7 10066552

[2] Gibson G, Wagner G. Canalization in evolutionary genetics: a stabilizing theory? BioEssays. 2000; 22: 372–80. 10.1002/(SICI)1521-1878(200004)22:4<372::AID-BIES7>3.0.CO;2-J 10723034

[3] West-Eberhard MJ. Developmental Plasticity and Evolution New York: Oxford University Press; 2003.

[4] Gibson G, Dworkin I. Uncovering cryptic genetic variation. Nat Rev Genet. 2004;5: 681–90. 10.1038/nrg1426 15372091

[5] DeWitt TJ, Scheiner SM. Phenotypic variation from single genotypes In: DeWitt TJ Scheiner SM, editors. Phenotypic Plasticity. Functional and Conceptual Approaches. Oxford: Oxford University Press; 2004 pp. 1–9.

[6] Jablonski D. The future of the fossil record. Science. 1999;284: 2114–6. 10.1126/science.284.5423.2114 10381868

[7] Palmer AR. Fluctuating asymmetry analyses: a primer In: Markow T, editor. Developmental Instability: Its Origins and Evolutionary Implications. Dordrecht: Kluwer; 1994 pp. 335–364.

[8] Willmore KE, Young NM, Richtsmeier JT. Phenotypic variability: its components, measurement and underlying developmental processes. Evol Biol. 2007;34: 99–120. 10.1007/s11692-007-9008-1

[9] Van Valen L. A study of fluctuating asymmetry. Evolution. 1962;16: 125–142. 10.2307/2406192

[10] Klingenberg CP. A developmental perspective on developmental instability: theory, models and mechanisms In: Polak M, editor. Developmental Instability: Causes and Consequences. New York, Oxford University Press; 2003 pp. 14–34.

[11] Willmore KE, Hallgrímsson B. Within individual variation: developmental noise versus developmental stability In: B. Hallgrímsson B, Hall BK, editors. Variation: A Central Concept in Biology. Burlington, MA: Elsevier; 2005 pp. 191–218.

[12] McAdams HH, Arkin A. Stochastic mechanisms in gene expression. Proc Natl Acad Sci. 1997;94: 814–819. 9023339 10.1073/pnas.94.3.814 PMC19596

[13] Kaern M, Elston TC, Blake WJ, Collins JJ. Stochasticity in gene expression: from theories to phenotypes. Nat Rev Genet. 2005;6: 451–64. 10.1038/nrg1615 15883588

[14] Lambert J, Thiéry PP. Essai de nomenclature raisonnée des échinides Chaumont: Librairie L. Ferrière; 1909–1925

[15] Stokes R. B. Royaumes et provinces fauniques du Crétacé établis sur la base d’une étude systématique du genre *Micraster*. Mem Mus Nat Hist Nat Nouv Ser. 1975;C 31: 1–94.

[16] Rowe AW. An analysis of the genus *Micraster*, as determined by rigid zonal collecting from the zone of *Rhynchonella Cuvieri* to that of *Micraster cor-anguinum*. Q J Geol Soc London. 1899; 55: 494–547. 10.1144/GSL.JGS.1899.055.01-04.34

[17] Ernst G. Zur Stammesgeschichte und stratigraphischen Bedeutung der Echiniden-Gattung *Micraster* in der nordwestdeutschen Oberkreide. Mitt Geol-Paläont Inst Hamburg. 1970;39: 117–135.

[18] Ernst G. Grundfragen der Stammesgeschichte bei irregulären Echiniden der nordwesteuropäischen Oberkreide. Geol Jb A. 1972;4: 63–175.

[19] David B, Fouray M. Variabilité et disjonction évolutive des caractères dans des populations de *Micraster* (Echinoidea, Spatangoidea) du Crétacé supérieur de Normandie. Geobios. 1984;17: 447–476.

[20] Smith AB, Wright CV. British Cretaceous echinoids. Part 9, Atelostomata, 2. Spatangoida (2). Palaeontogr Soc Monogr. 2012;166: 635–754.

[21] Drummond PVO. The *Micraster* biostratigraphy of the Senonian white chalk of Sussex, southern England. Geol Mediterr. 1983;10: 177–182.

[22] Ernst G, Seibertz E. Concepts and methods of echinoid biostratigraphy In: Kauffman EG, Hazel JE, editors. Concepts and Methods of Biostratigraphy. Stroudsburg: Hutchinson & Ross; 1977 pp. 541–563.

[23] Smith AB. Echinoid Palaeobiology. London: Allen & Unwin; 1984.

[24] Olszewska-Nejbert D. Late Cretaceous (Turonian—Coniacian) irregular echinoids of western Kazakhstan (Mangyshlak) and southern Poland (Opole). Acta Geol Pol. 2007;57: 1–87.

[25] Villier L, David B, Néraudeau D. Ontogenetic and morphological evolution of the ambulacral pores in *Heteraster* (early spatangoids) In: Barker M, editor. Echinoderms 2000. Lisse: Swets & Zeitlinger; 2001 pp. 563–567.

[26] Wiese F. Das Turon und Unter-Coniac im Nordkantabrischen Becken (Provinz Kantabrien, Nordspanien): Faziesentwicklung, Bio-, Event- und Sequenzstratigraphie. Berliner geowiss Abh E. 1997;24: 1–131.

[27] Seibertz E. Stratigraphie, Fazies und Paläogeographie der „Mittel"-Kreide zwischen Rüthen und Erwitte (Alb-Coniac, SE-Münsterland). Aufschluß Sonderbd. 1979;29: 85–92.

[28] Kaplan U, Skupin K. Coniacian near Erwitte. In: Mutterlose J; Bornemann A, Rauer S; Spaeth C, Wood CJ, editors. Key localities of the northwest European Cretaceous. Bochumer geol u geotechn Arb. 1998;48: 184–185.

[29] Voigt S, Wilmsen M, Mortimore RN, Voigt T. Cenomanian paleotemperatures derived from the oxygen isotopic composition of brachiopods and belemnites: evaluation of Cretaceous paleotemperature proxies. Int J Earth Sci. 2003;92: 285–299. 10.1007/s00531-003-0315-1

[30] Ziegler PA. Evolution of the Arctic-North Atlantic and the Western Tethys. Am Assoc Pet Geol Mem. 1988;43: 198 pp.

[31] Higgins R. C. Specific status of *Echinocardium cordatum*, *E*. *australe* and *E*. *zealandicum* (Echinoidea: Spatangoida) around New Zealand, with comments on the relation of morphological variation to environment. J Zool. 1974;173: 451–47. 10.1111/j.1469-7998.1974.tb04127.x

[32] Zaghbib-Turki D. Stratégie adaptive des *Hemiaster* et des *Periaster* du Crétacé supérieur (Cénomanien-Coniacien) de la Plate-forme carbonatée de Tunisie In: De Ridder C, Dubois P, Lahaye MC, Jangoux M, editors. Echinoderm Research. Rotterdam: Balkema; 1990 pp. 49–56.

[33] Kanazawa K. Adaptation of test shape for burrowing and locomotion in spatangoid echinoids. Palaeontology. 1992;35: 733–750.

[34] François É, David B. Variations morphologiques des *Toxaster* (Echinoida: Spatangoida) en regard des fluctuations spatiales (Arc de Castellane, SE France) et temporelles (Valanginien-Hauterivien) du milieu sédimentaire: expression d’un potentiel adaptatif restreint. Geobios. 2006;39: 355–371. 10.1016/j.geobios.2005.01.002

[35] Saitoh M, Kanazawa K. Adaptive morphology for living in shallow water environments in spatangoid echinoids. In: Kroh A, Reich M, editors. Echinoderm Research 2010: Proceedings of the Seventh European Conference on Echinoderms, Göttingen, Germany, 2–9 October 2010. Zoosymposia. 2012;7: 255–265.

[36] Nichols D. Changes in the chalk heart-urchin *Micraster* interpreted in relation to living forms. Philos Trans R Soc Lond B Biol Sci. 1959;242: 347–437. 10.1098/rstb.1959.0007

[37] Fouray M. L’evolution des *Micraster* (Echinides, Spatangoida) dans le Turonien-Coniacien de Picardie occidentale (Somme). Interets biostratigraphiques. Ann Palaeontol (Invert). 1981;67: 81–134.

[38] Smith AB. The structure, function and evolution of tube feet and ambulacral pores in irregular echinoids. Palaeontology. 1980;23: 39–83.

[39] Kaplan U, Kennedy WJ. Die Ammoniten des westfälischen Coniac. Geol Palaeont Westf. 1994;31: 1–155.

[40] Tröger K-A. Zur Biostratigraphie des Ob.-Turons bis Unt.-Santons aus dem Schachtaufschluß der Zeche Grimberg IV bei Bergkamen (BRD). Freiberg Forschungsh C. 1974;298: 109–138.

[41] Falkingham PL. Acquisition of high resolution 3D models using free, open-source, photogrammetric software. Palaeontol Electron. 2012;15: 15p. palaeo-electronica.org/content/issue-1-2012-technical-articles/92-3d-photogrammetry

[42] Mallison H, Wings O. Photogrammetry in paleontology–a practical guide. J Paleontol Tech. 2014;12: 1–3.

[43] Klingenberg CP. MorphoJ: an integrated software package for geometric morphometrics. Mol Ecol Resour. 2010;11: 353–357. 10.1111/j.1755-0998.2010.02924.x 21429143

[44] Bookstein FL. Morphometric tools for landmark data: geometry and biology Cambridge: Cambridge University Press; 1991.

[45] Klingenberg CP, McIntyre GS. Analyzing patterns of fluctuating asymmetry with Procrustes methods. Evolution. 1998;52: 1363–1375. 28565401 10.1111/j.1558-5646.1998.tb02018.x

[46] Klingenberg CP, Barluenga M, Meyer A. Shape analysis of symmetrical structures: quantifying variation among individuals and asymmetry. Evolution. 2002;56: 1909–1920. 12449478 10.1111/j.0014-3820.2002.tb00117.x

[47] Néraudeau D, David B, Madon C. Tuberculation in spatangoid fascioles: Delineating plausible homologies. Lethaia. 1998;31: 323–334.

[48] Schneider CA, Rasband WS, Eliceiri KW. NIH Image to ImageJ: 25 years of image analysis. Nat Methods. 2012;9: 671–675. 22930834 10.1038/nmeth.2089 PMC5554542

[49] Palmer AR, Strobeck C. Fluctuating asymmetry: measurement, analysis, patterns. Annu Rev Ecol Syst. 1986;17: 391–421. 10.1146/annurev.es.17.110186.002135

[50] Palmer AR, Strobeck C. Fluctuating asymmetry revisited In: Polak M, editor. Developmental Instability (DI): Causes and Consequences. Oxford: Oxford University Press; 2003 pp. 279–319.

[51] Swain DP. A Problem with the use of meristic characters to estimate developmental stability. Am Nat. 1987;129: 761–768.

[52] Walker DE, Gagnon J-M. Locomotion and functional spine morphology of the heart urchin *Brisaster fragilis*, with comparisons to *B*. *latifrons*. J Mar Biol. 2014;Article ID 297631, 9 pages. 10.1155/2014/297631

[53] Egea E, David B, Choné T, Laurin B, Féral JP, Chenuil A. Morphological and genetic analyses reveal a cryptic species complex in the echinoid *Echinocardium cordatum* and rule out a stabilizing selection explanation. Mol Phylogenet Evol. 2015;94: 207–220. 10.1016/j.ympev.2015.07.023 26265259

[54] De Baets K, Bert D, Hoffmann R, Monnet C, Yacobucci MM, Klug C. Chapter 9. Ammonoid intraspecific variability. In: Klug C, Korn D, De Baets K, Kruta I, Mapes RH, editors. Ammonoid Paleobiology: From Anatomy to Ecology. Topics In Geobiology. 2015;43: 359–426.

[55] McKinney ML, McNamara KJ. Heterochrony. The Evolution of Ontogeny. New York, London: Plenum Press; 1991.

[56] Chauffe KM, Nichols PA. Differentiating evolution from environmentally induced modifications in mid-Carboniferous conodonts. Palaeontology. 1995;38: 875–895.

[57] Scheiner SM. Genetics and Evolution of Plasticity. Annu Rev Ecol Syst. 1993;24: 35–68.

[58] Braendle C, Flatt T. A role for genetic accommodation in evolution? BioEssays. 2006;28: 868–873. 16937342 10.1002/bies.20456

[59] Suzuki Y, Nijhout HF. Evolution of a polyphenism by genetic accommodation. Science. 2006;311: 650–652. 16456077 10.1126/science.1118888

[60] Cunningham JA, Jeffery Abt CH. Coordinated shifts to non-planktotrophic development in spatangoid echinoids during the Late Cretaceous. Biol Lett. 2009;5: 647–650. 10.1098/rsbl.2009.0302 19515650 PMC2781954

[61] Wiese F, Voigt S. Late Turonian (Cretaceous) climate cooling in Europe: faunal response and possible causes. Geobios. 2002;35: 65–77. 10.1016/S0016-6995(02)00010-4

[62] Chenuil A, Féral JP. Sequences of mitochondrial DNA suggest that *Echinocardium cordatum* is a complex of several sympatric or hybridizing species. A pilot study. In: Féral J-P, David B, editors. Echinoderm Research 2001: proceedings of the 6th European Conference on Echinoderm Research, Banyuls-sur-mer, 3–7 September 2001. Lisse: Swets & Zeitlinger; 2003. pp. 15–21.

[63] Klingenberg CP, Nijhout HF. Genetics of fluctuating asymmetry: a developmental model of developmental instability. Evolution. 1999;53: 358–375. 10.2307/2640773 28565420

[64] Hunt G. Phenotypic variation in fossil samples: modeling the consequences of time-averaging. Paleobiology. 2004;30: 426–443. 10.1666/0094-8373(2004)030<0426:PVIFSM>2.0.CO;2

[65] Chalancon G, Ravarani CNJ, Balaji S, Martinez-arias A, Aravind L, Jothi R, Madan Babu M. Interplay between gene expression noise and regulatory network architecture. Trends Genet. 2012;28: 221–232. 10.1016/j.tig.2012.01.006 22365642 PMC3340541

[66] Arthur W. The concept of developmental reprogramming and the quest for an inclusive theory of evolutionary mechanisms. Evol Dev. 2000;2: 49–57. 10.1046/j.1525-142X.2000.00028.x 11256417

[67] McNamara KJ. Plate translocation in spatangoid echinoids: its morphological, functional and phylogenetic significance. Paleobiology. 1987;13: 312–325.

[68] Saucède T, Alibert P, Laurin B, David B. Environmental and ontogenetic constraints on developmental stability in the spatangoid sea urchin *Echinocardium* (Echinoidea). Biol J Linn Soc. 2006;88: 165–177.

[69] Lawrence JM, Pomory CM, Sonnenholzner J, Chao C-M. Bilateral symmetry of the petals in *Mellita tenuis*, *Encope micropora*, and *Arachnoides placenta* (Echinodermata: Clypeasteroida). Invert Biol. 1998;117: 94–100. 10.2307/3226855

[70] Stige LC, David B, Alibert P. On hidden heterogeneity in directional asymmetry—can systematic bias be avoided? J Evol Biol. 2006;19: 492–499. 10.1111/j.1420-9101.2005.01011.x 16599925

[71] Néraudeau D. La variabilité morphologique est-elle un obstacle à la définition des espèces paléontologiques? Le cas des échinides spatangues, Biosystema. 2001;19: 93–107.

[72] De Ridder C, Lawrence J. Food and feeding mechanisms: Echinoidea In: Jangoux M, Lawrence J, editors. Echinoderm Nutrition. Rotterdam: A.A. Balkema Press; 1982 pp. 97–115.

[73] McNamara KJ. Philip GM. Australian tertiary schizasterid echinoids. Alcheringa. 1980;4: 47–65. 10.1080/03115518008558980

[74] Møller AP, Swaddle JP. Asymmetry, Developmental Stability, and Evolution Oxford: Oxford University Press; 1997.

[75] Karvonen E, Merilä J, Rintamäki PT, Van Dongen S. Geography of fluctuating asymmetry in the greenfinch, *Carduelis chloris*. Oikos. 2003;100: 507–516.

[76] Polak M. Ectoparasitism in mothers causes higher positional fluctuating asymmetry in their sons : implications for sexual selection. Am Nat. 1997;149: 955–974. 18811257 10.1086/286032

