# Supplementary figures and images for: Ecophenotypic Variation and Developmental Instability in the Late Cretaceous Echinoid Micraster brevis (Irregularia; Spatangoida)
Source: PLoS One. 2016 Feb 5;11(2):e0148341. doi: 10.1371/journal.pone.0148341 (PMC4746069; doi:10.1371/journal.pone.0148341)

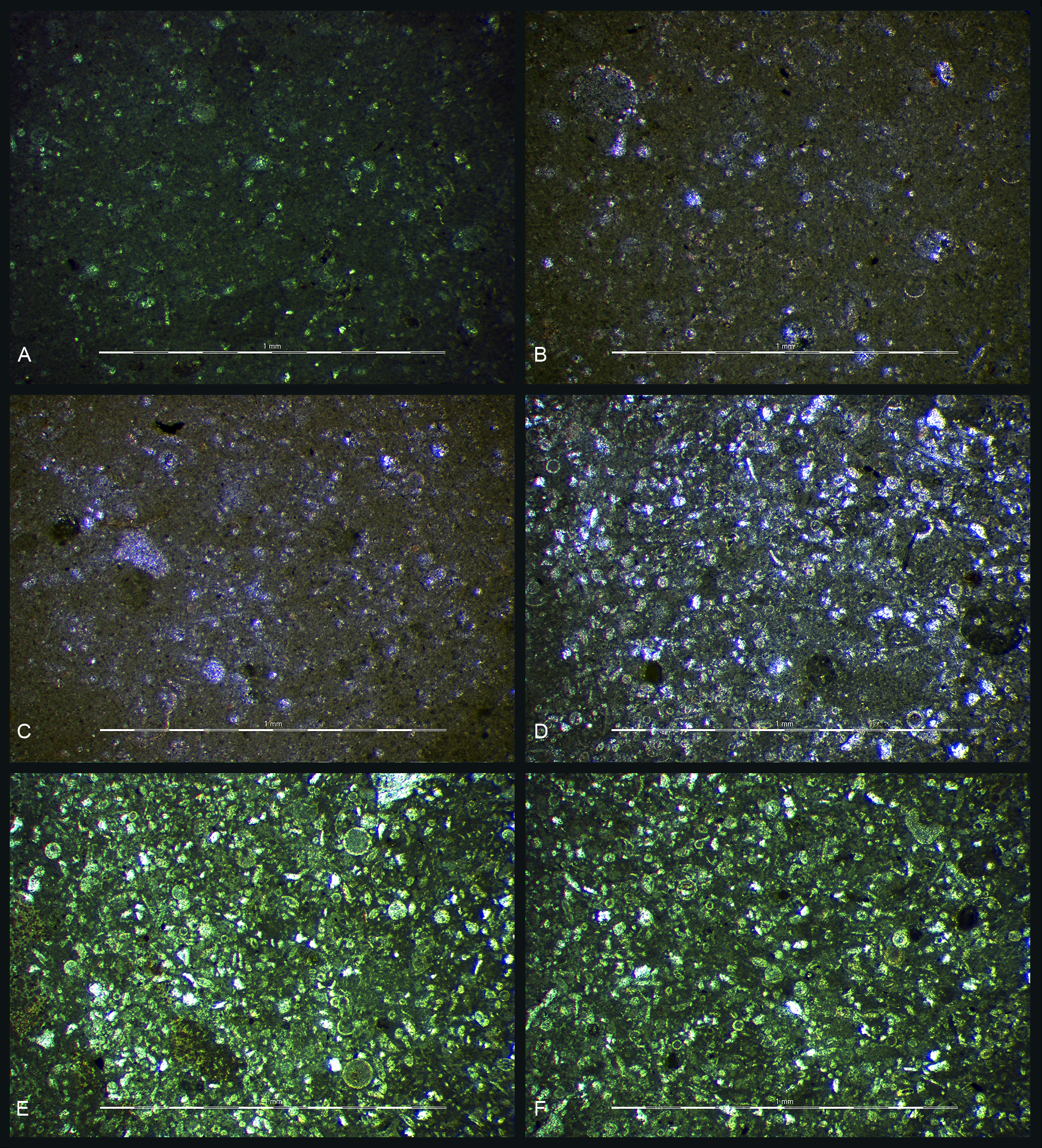

Supplement: S1 Fig — (A) Grimberg. (B, C) Erwitte. (D-F) Liencres. (A-C) Wackestones contain clay and silt, with relatively low content of bioclasts. (D-F) Silty packstones with abundant bioclasts and siliciclasts. (TIF) [file pone.0148341.s001.tif]

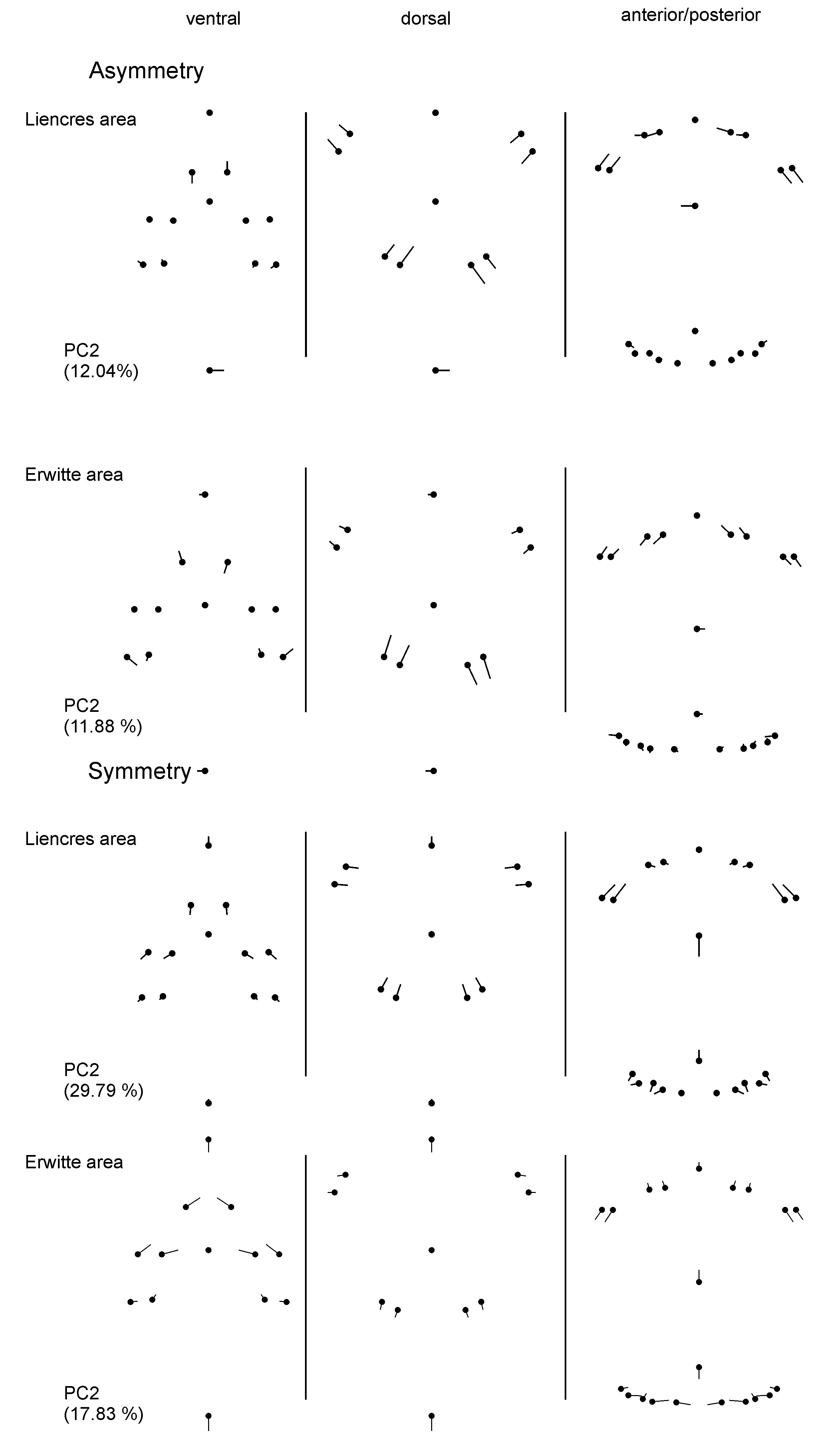

Supplement: S2 Fig — (TIF) [file pone.0148341.s002.tif]

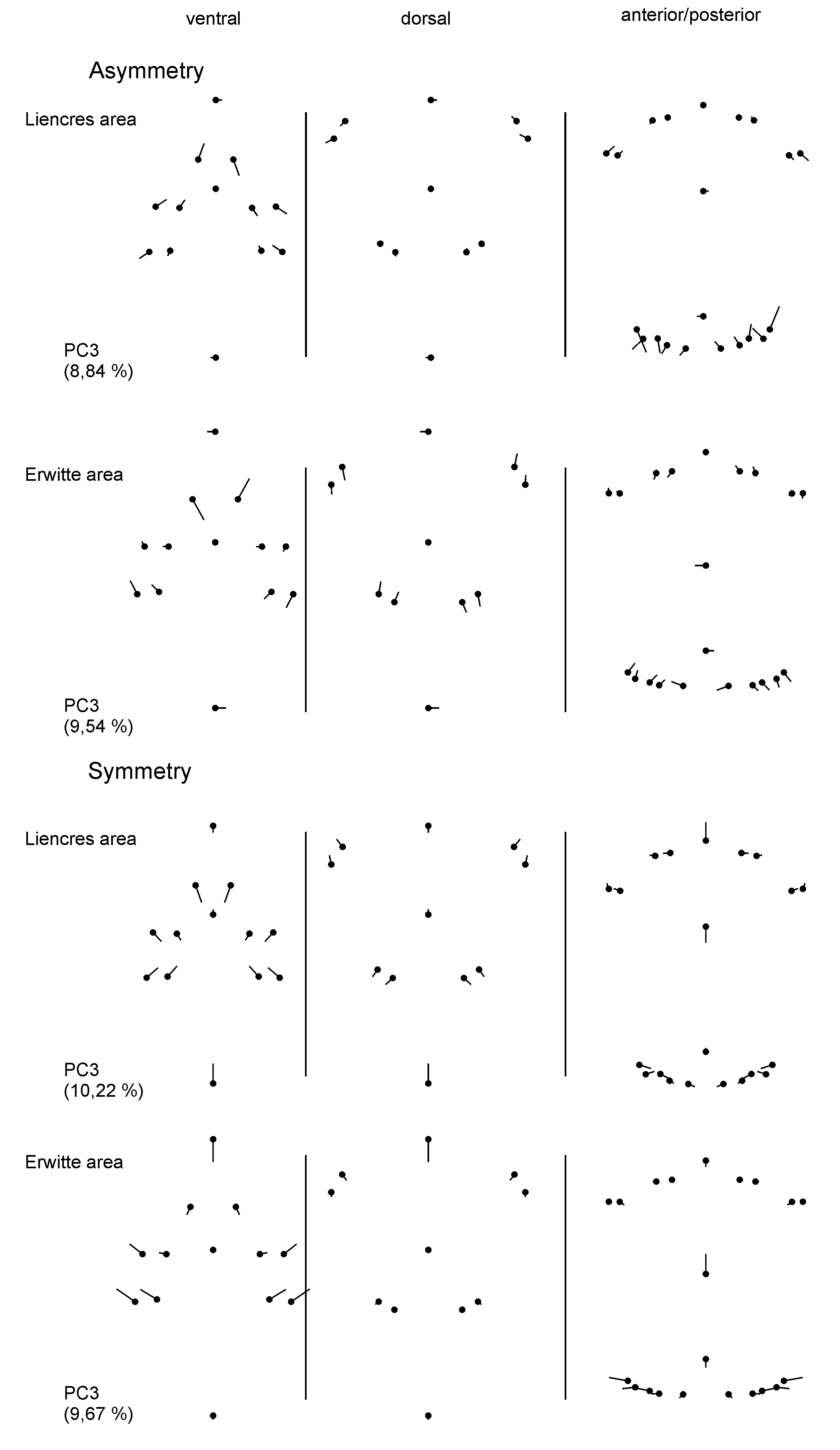

Supplement: S3 Fig — (TIF) [file pone.0148341.s003.tif]

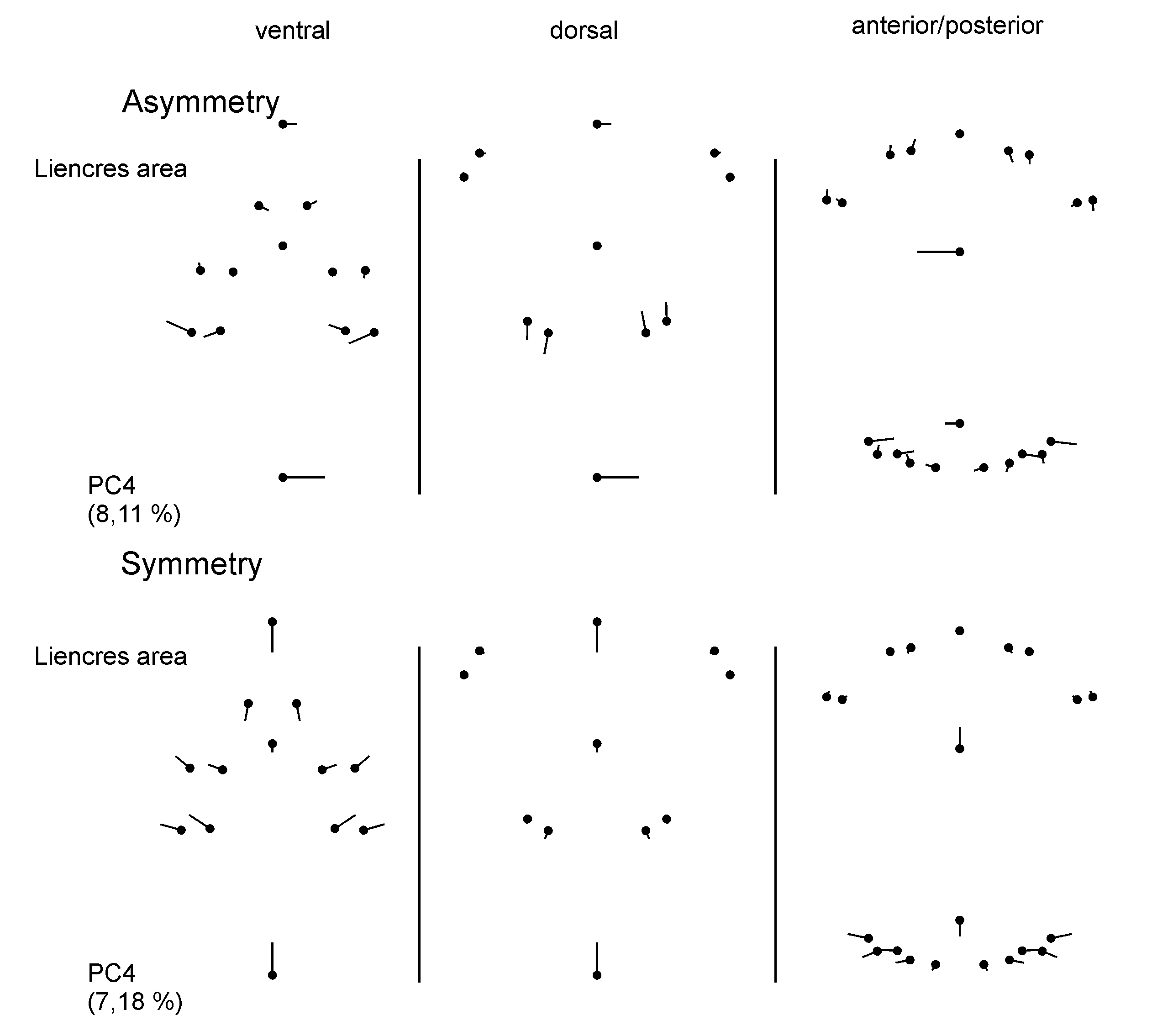

Supplement: S4 Fig — (TIF) [file pone.0148341.s004.tif]

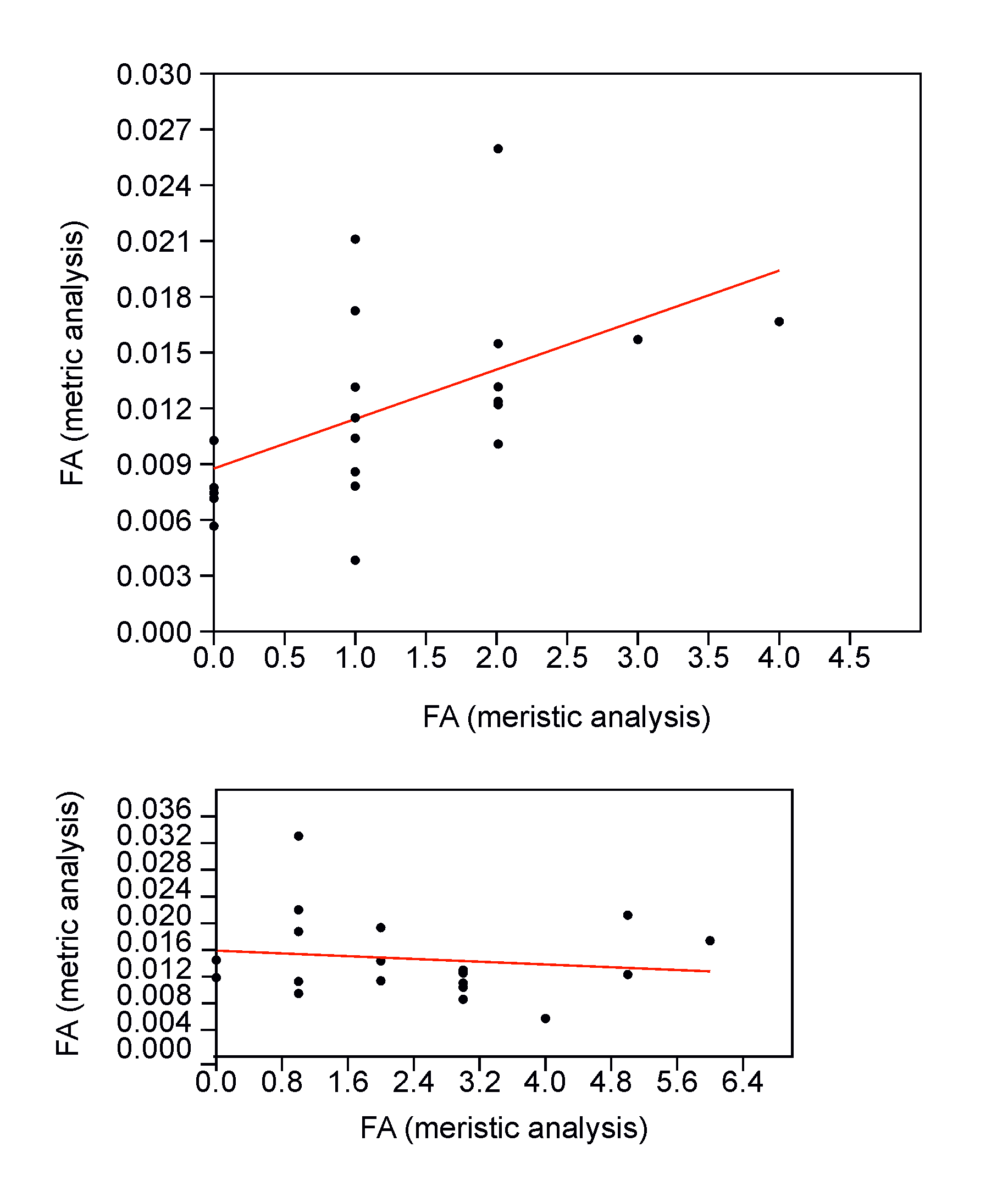

Supplement: S5 Fig — Comparison of the individual metric and meristic FA values for the specimens from Liencres area (A) and from the Erwitte area (B). (TIF) [file pone.0148341.s005.tif]

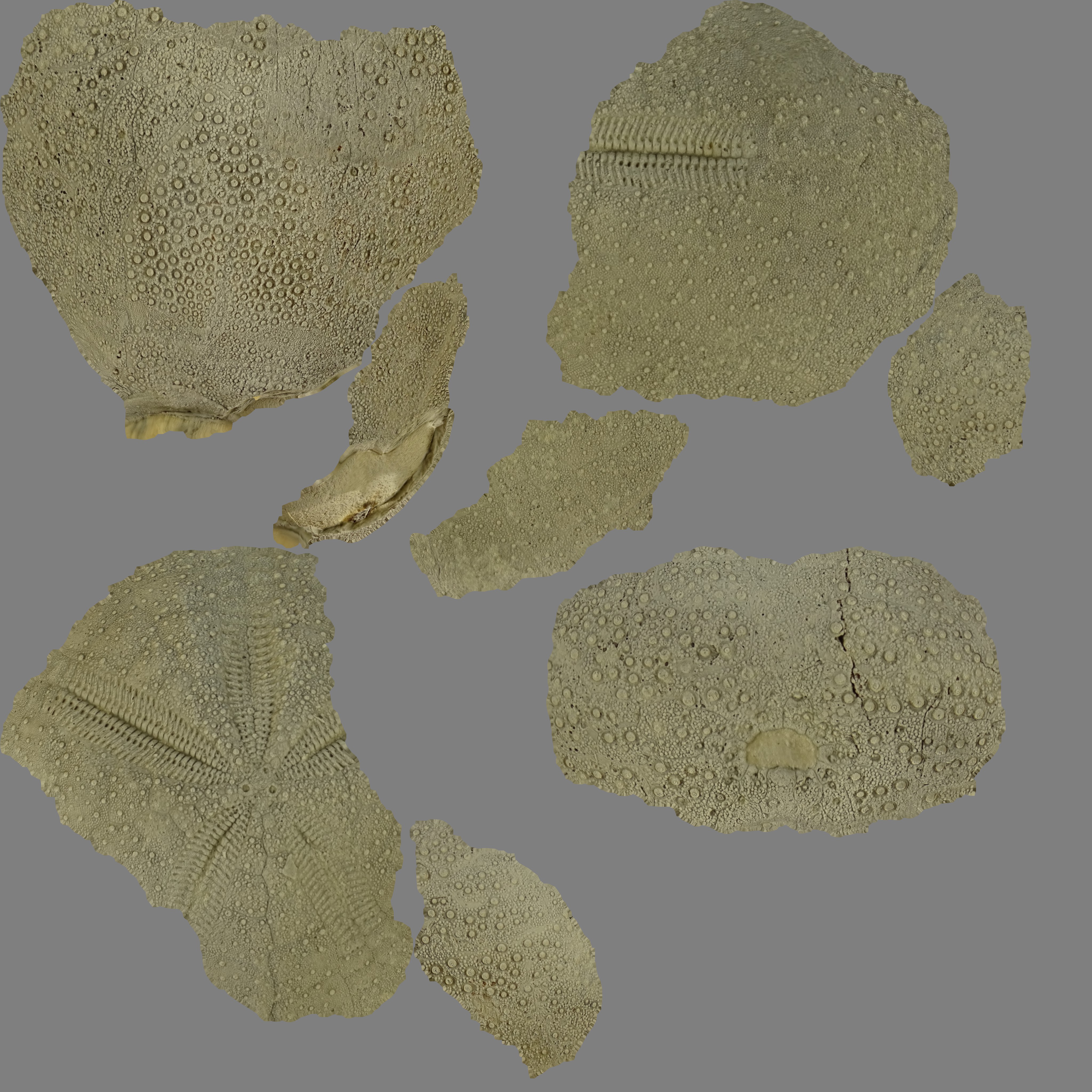

Supplement: S1 Multimedia — This file (.obj file)can be opened (import mesh) in the open-source software MeshLab (Visual Computing Lab—ISTI—CNR), available at: http://meshlab.sourceforge.net/. The landmark coordinates (GSUB E3840_picked_points.pp) can be loaded via the PickPoints function. (ZIP) [file pone.0148341.s007.zip › landmark setting_GSUB E3840/GSUB_E3840_tex_0.jpg]

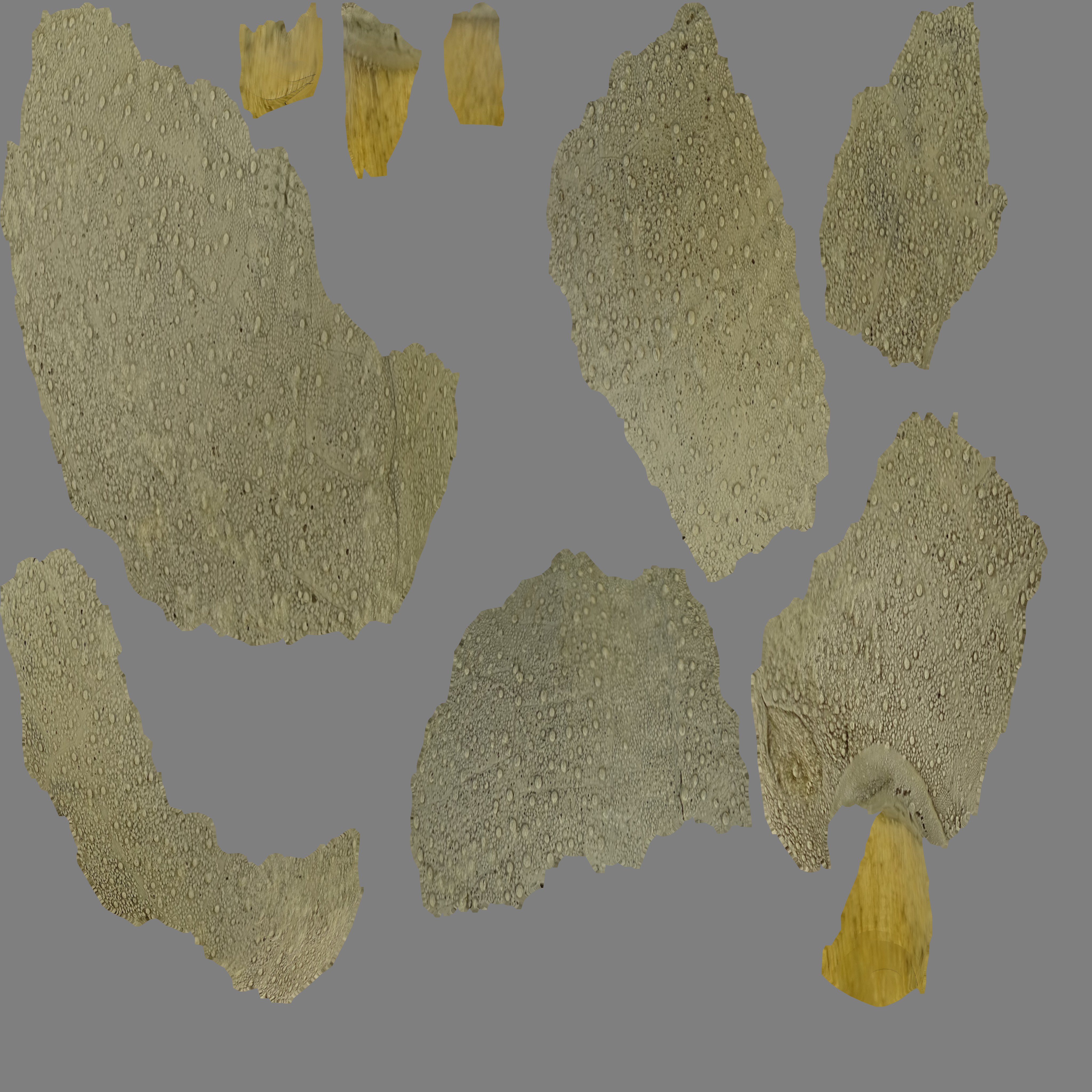

Supplement: S1 Multimedia — This file (.obj file)can be opened (import mesh) in the open-source software MeshLab (Visual Computing Lab—ISTI—CNR), available at: http://meshlab.sourceforge.net/. The landmark coordinates (GSUB E3840_picked_points.pp) can be loaded via the PickPoints function. (ZIP) [file pone.0148341.s007.zip › landmark setting_GSUB E3840/GSUB_E3840_tex_1.jpg]
