# Supplementary material for: Ecophenotypic Variation and Developmental Instability in the Late Cretaceous Echinoid Micraster brevis (Irregularia; Spatangoida)
Source: PLoS One. 2016 Feb 5;11(2):e0148341. doi: 10.1371/journal.pone.0148341 (PMC4746069; doi:10.1371/journal.pone.0148341)
Supplement: S1 File — (DOC) [file pone.0148341.s006.doc]

**Morphometric variation**

**Shape analysis**

*Grimberg IV shaft*: Kr 47601 – 06, Kr 4768 – 13, Kr 476015, X 06195

*Erwitte area*: GSUB E3812 – 20, E3822, E3825 – 28, E3839 – 41, E3843 – 61

*Liencres area*: EE4485, MBE3870, 3872, 8008, 8190, 8194, 8196 – 97, 8200, 8231, 8234, 8240, 8245, 8247 – 48, 8251, 8254, 8258 – 59, 8261, 8264, 8266, 8270, 8274 – 75, 8304, 8309, 8310, 8320, 8326 – 27, 8328, 8399, 8400, 8458

**Fluctuating asymmetry analysis**

*Erwitte area*: GSUB E3812 - 15, E3817 - 19, E3821 - 22, E3825 - 27 E3839 - 61

*Liencres area*: EE4485, MBE3870, 3872 – 73, 8008, 8190, 8191, 8194, 8196 – 97, 8200, 8231, 8234, 8240, 8245, 8247 – 48, 8251, 8254, 8258 – 59, 8261, 8265 – 66, 8270, 8274 – 75, 8304, 8308, 8309, 8310, 8320, 8326 – 28, 8399, 8400, 8458

**Non-morphometric variation**

**Variation in the subanalfasciole**

*Grimberg IV shaft*: Kr 47601 – 17, 47619 – 26, X 06195

*Erwitte area*: GSUB E3812 –61

*Liencres area*: MBE3870, 3873, 8008, 8190, 8191, 8194, 8196 – 97, 8199, 8200, 8202, 8228, 8231, 8234, 8237, 8240, 8241, 8245, 8247 – 48, 8251, 8254 – 55, 8257 – 59, 8261, 8264, 8265 – 66, 8279, 8274 – 75, 8304, 8308, 8309, 8310, 8316, 8320, 8322, 8326 – 27, 8328, 8399, 8400, 8458

**Projection of the labrum,**

*Grimberg IV shaft*: Kr 47601, Kr 47603 – 06, Kr 47611, Kr 47613 , Kr 47616 – 18, Kr 47620 - 26

*Erwitte area*: GSUB E3812 – 13, E3815 – 16, E3818 – 22, E3824 - 41, E3843 - 61

*Liencres area*: MBE3873, 8008, 8190, 8191, 8194, 8196, 8199, 8200, 8202, 8228, 8231, 8234, 8237, 8238, 8240, 8245, 8247 – 48, 8251, 8254 – 55, 8257 – 59, 8261, 8264, 8266, 8279, 8308, 8327, 8399, 8400, 8458

**Interradial structure of the paired petals and granulation of the periplastronal area**

*Grimberg IV shaft*: Kr 47601 – 47626

*Erwitte area*: GSUB E3812 –61

*Liencres area*: EE4485, MBE3870, 3872 – 73, 8008, 8190, 8191, 8194, 8196 – 97, 8199, 8200, 8202, 8228, 8231, 8234, 8237, 8238, 8240, 8241, 8245, 8247 – 48, 8251, 8254 – 55, 8257 – 59, 8261, 8264, 8265 – 66, 8279, 8274 – 75, 8304, 8308, 8309, 8310, 8316, 8320, 8322, 8326 – 27, 8328, 8399, 8400, 8458

**Variations in pore pair numbers of the paired petals / FA analyses in pore pair numbers of the paired petals**

*Erwitte area:* GSUB E3812 - 14, E3816 - 21, E3823 - 26. E3828, E3831, E3839, E3841 - 45, E3847 - 52, E3854 - 57, E3859, 63, E3866 - 67

*Liencres area*: EE4485, MBE3873, 3875, 8008 - 09, 8191, 8194, 8202, 8228, 8234, 8237, 8238, 8241, 8247 – 48, 8251, 8254, 8258 – 60, 8265, 8270, 8273, 8275 – 76, 8304, 8309 - 10, 8316, 8318, 8320, 8324, 8326, 8329, 8400, 8458
